# A network meta-analysis of maintenance therapy in chronic lymphocytic leukemia

**DOI:** 10.1371/journal.pone.0226879

**Published:** 2020-01-29

**Authors:** Cho-Hao Lee, Po-Huang Chen, Chin Lin, Chieh-Yung Wang, Ching-Liang Ho

**Affiliations:** 1 Division of Hematology and Oncology Medicine, Department of Internal Medicine, Tri-Service General Hospital, National Defense Medical Center, Taipei, Taiwan, ROC; 2 School of Public Health, National Defense Medical Center, Taipei, Taiwan, ROC; 3 Department of Research and Development, National Defense Medical Center, Taipei, Taiwan, ROC; 4 Division of Pulmonary and Critical Care Medicine, Department of Internal Medicine, Tri-Service General Hospital, National Defense Medical Center, Taipei, Taiwan, R.O.C; University of Manitoba, CANADA

## Abstract

**Background:**

Chronic lymphocytic leukemia (CLL) is incurable through conventional chemoimmunotherapy regimens. Despite durable responses to front-line therapy and sustained remission rates in patients with CLL, a majority of patients eventually relapse in 5 years of initial treatment. The depth of the response may affect the length of response. Maintenance therapies were aimed to deep remissions and extend the period of disease quiescence. Lenalidomide, rituximab and ofatumumab had demonstrated some efficacy as a maintenance therapy compared to no intervention for CLL patients. The relative effect on disease control and safety between different maintenance therapies were unclear.

**Methods:**

We performed a systematic literature review and network meta-analysis to evaluate relative effect on disease control and safety of current available maintenance therapies. We searched PubMed, Embase and Cochrane database up to March 6, 2019. Relevant reference of review article and conference abstract including European Hematology Association Annual Meeting (EHA 2018), American Society of Hematology Annual Meeting (ASH 2018) and American Society of Clinical Oncology Annual Meeting (ASCO 2018) were searched. Randomized controlled trials (RCT) involving current available maintenance therapy including “Lenalidomide”, “Rituximab”, “Ofatumumab”, “Ibrutinib”, “Idelalisib”, “Venetoclax”and “Obinutuzumab”were eligible. Outcomes of interest included progression-free survival (PFS), overall survival (OS) and serious adverse events (SAE) in CLL patients received subsequent maintenance therapy. Two authors CHL and CL) independently assessed eligibility for all identified citations and extracted data from the original trial reports. The selected studies’ risk of bias was assessed following the guidelines of Cochrane Collaboration Handbook.

**Results:**

In total, six phase III RCTs with total 1,615 CLL patients were identified. Maintenance therapy using lenalidomide, rituximab, and ofatumumab demonstrated a statistically significant effect in prolongation of progression-free survival (HR:0.37, 95% CI: 0.27–0.50 of lenalidomide; HR:0.50, 95% CI: 0.38–0.66 of rituximab; HR:0.52, 95% CI:0.41–0.66 of ofatumumab, separately) compared with no intervention; however, for overall survival, the effect of maintenance therapy showed no significant difference versus no intervention (HR: 0.89, 95% CI: 0.70–1.14). Lenalidomide showed the best efficacy for PFS (HR: 0.37, 95% CI: 0.27–0.50, Probability of being best treatment: 96%).

**Conclusions:**

Our network meta-analysis provided an integrated overview of relative efficacy and safety of different maintenance therapies in CLL. All maintenance therapies were effective in reducing the risk of disease progression versus no intervention. Based on current best evidence, maintenance therapy with lenalidomide is the most efficacious option.

## 1. Introduction

Chronic lymphocytic leukemia (CLL) is the most common lymphoproliferative disease in adults (median age: 72 years) [1] with the age-adjusted incidence being five per 1 hundred thousand person years [2, 3]. CLL has a variable clinical course and overall survival (OS) ranges from 18 months to more than 20 years [1, 4].

In the last few decades, to predict prognosis and assist in treatment planning, the CLL international prognostic index (CLL IPI) has been developed. Five important prognostic factors have been identified, namely, TP53 status, immunoglobulin heavy chain (IGHV) mutational status, serum beta2-microglobulin concentration, clinical stage, and age [5]. Moreover, there are different types of chemotherapy and immunotherapy regimens that prolong PFS, OS and remission rates; however, CLL remains incurable through conventional chemoimmunotherapy regimens. Despite durable responses to front-line therapy and sustained remission rates in patients with CLL, a majority of patients eventually relapse in 5 years of initial treatment [6, 7]. Moreover, the risk of relapse increases in patients characterized by high-risk features (such as unmutated IGHV, TP53 mutation, del[17p]) or those with disease refractory to therapy [8–11]. Generally, these patients experience early progression of the disease and salvage therapies are not sufficiently effective, leading to a significantly reduced OS [12].

For patients that require second-line treatment, the national comprehensive cancer network (NCCN) guidelines recommend re-treatment with a regimen that was used for first-line therapy or lenalidomide for high-risk patients (ie. ummutated IGHV) [13]. However, when used in previously treated patients, fludarabine, cyclophosphamide and rituximab (FCR) yields few complete responses and short remission times [6, 14, 15]. Moreover, second-line treatment with targeted therapies, notably B-cell receptor inhibitors (ibrutinib and idelalisib) or Bcl-2 inhibitor (venetoclax), leads to partial and sustained response in most patients that have relapsed or have refractory disease. However, achieving a complete response without minimal residual disease is rare [16–18].

Given the progressive shortening of PFS period after each line of therapy, there is a requirement for newer therapeutic strategies such as maintenance therapy. Accumulating evidence for other hematological malignancies (i.e., multiple myeloma or diffuse large B-cell lymphoma) demonstrated that maintenance therapy with lenalidomide prolongs PFS [19, 20]. Given this context, it has been suggested that maintenance therapy after chemoimmunotherapy may prolong remission duration by preventing reemergence of malignant CLL clones [21, 22] and improve the extent and duration of response to initial therapies. Thus, maintenance strategies could be considered for patients that have high-risk CLL [23].

In 2015, van Oers et al. [24] reported a significant improvement in median PFS (ofatumumab:29.4 months vs No intervention:15.2 months, P<0.0001) after administrating maintenance therapy using ofatumumab in patients with relapsed CLL in remission. After this pilot trial, a number of maintenance therapy studies were conducted. In 2017, the CLLM1 [25] and CONTINUUM [26] trials reported a superior effect of maintenance therapy using lenalidomide compared with no intervention. CLLM1 trial included 89 CLL patients after first-line chemoimmunotherapy and reported statistically significant improvement in prolongation of PFS (HR:0·168, 95% CI:0.074–0.379, P<0.0001) [25]. CONTINUUM trial reported more favorable effect versus no intervention in reduction risk of disease progression (HR:0.40, 95% CI:0.29–0.55; P<0·0001) in 314 previously treated CLL patients [26]. Moreover, the benefits of rituximab maintenance compared with no intervention were demonstrated in the CLL-2007-SA [27] and AGMT CLL-8a [28] trials. CLL-2007-SA trial recruited 409 previously untreated CLL patients and compared rituximab to no intervention which demonstrated that 2-year maintenance rituximab achieved better PFS (HR:0.55, 95%CI:0.40–0.75, P<0.001) with acceptable safety profile [27]. 263 CLL patients who respond to first-line or second-line rituximab-containing chemoimmunotherapy were included in AGMT CLL-8a trial, the results showed PFS was statistically significant longer in rituximab maintenance group than no intervention (HR:0.50, 95% CI:0.33–0.75, P<0.001) [28]. Furthermore, in 2017, the GALACTIC [29] trial started to evaluate the effect of maintenance therapy using a new anti-CD20 monoclonal antibody obinutuzumab.

Thus, although maintenance therapies showed effects to prolong PFS versus no intervention, relative efficacy and safety between different treatment options remains unclear. The approach to selecting a maintenance treatment regimen is not conclusive because of the lack of head-to-head direct comparison studies. Also, clinical decision-making for selecting maintenance therapy is judged by factors of response of front-line therapies, the presence of high-risk features, and patient fitness [9, 30] without an integrated evidence for reference.

In fact, currently, there is still an unmet requirement for improving clinically relevant outcomes for previously treated patients with CLL. Therefore, to assess the relative efficacy and safety of different maintenance therapies in patients with CLL, we performed a systematic review and network meta-analysis (NMA) of all available phase III RCTs.

## 2. Materials and methods

### 2.1. Search strategy

We performed a systematic review and NMA and examined the Cochrane Central Register of Controlled Trials, Embase, LILACS database, PubMed, the UK National Research Register, CNKI, Google Scholar, and Clinicaltrial.gov right from their initial trials to those registered up to Sep 6, 2019, without language restrictions. We also searched relevant reference of review article and conference abstract including European Hematology Association Annual Meeting (EHA 2019), American Society of Hematology Annual Meeting (ASH 2018) and American Society of Clinical Oncology Annual Meeting (ASCO 2019).

The search terms comprised of “Chronic lymphocytic leukemia” and “Maintenance” along with a list of all interventions and possible relevant key words (S1 Appendix). Our systematic review was performed based on the guidelines and recommendations stated in the preferred reporting items for systematic reviews and meta-analyzes (PRISMA) checklist (S2 Appendix) [31, 32].

### 2.2. Eligibility criteria

The selected studies involved patients who had a definitive diagnosis of CLL who had achieved at least a partial response after previous treatment and received subsequent maintenance therapy. Diagnosis of CLL was determined based on the criteria established by the International Workshop on Chronic Lymphocytic Leukemia [33]. Current available and developing maintenance therapies were involved including “Lenalidomide”, “Rituximab”, “Ofatumumab”, “Ibrutinib”, “Idelalisib”, “Venetoclax”and “Obinutuzumab“. Studies were eligible if they were randomized controlled trials (RCTs) which compared above maintenance agents. Also, RCTs compared maintenance agent with either placebo or observation were included. No language limitation was adopted. The primary outcome of the study was OS. Secondary outcomes were PFS and serious adverse events (SAE). PFS was defined as time from the date of randomization to the date of progression of disease, relapse or death from any cause. SAE was comprised of grade 3 and 4 adverse events defined by Common Terminology Criteria for Adverse Events (CTCAE) [34]. We excluded studies if they were not reporting outcomes of interest.

### 2.3. Study selection

Two authors (CHL and PHC) independently screened the abstracts and titles of eligible publications and judged whether to further review the full text. We emailed the trial author when full texts were unavailable. Full texts were independently reviewed by CHL and PHC. In the case of any disagreement during the selection process, the decisions were obtained after group discussion. Finally, we used PRISMA flow chart to show the total number of retrieved references and the number of included and excluded studies.

### 2.4. Data extraction

Two reviewers (CHL and PHC) independently extracted data from original trial reports by using a specifically designed form. We captured information for study characteristics (trial names, registration code of NCT, publication year, study design, follow-up duration); patient characteristics (inclusion criteria, mean age, percentage of patients who received first-line treatment, first-line regimen); sample sizes; and details of interventions with comparisons and outcomes.

For time to event outcomes (PFS and OS), we extracted hazard ratios, standard errors and the number of randomized patients; for dichotomous outcomes (SAE), we extracted the number of patients randomized, the number of patients analyzed and the number of event per arm. If trials reported outcomes both from per-protocol and intention-to-treat (ITT) design, we extracted the data from ITT design. For the analysis, if the HR with standard error data was incomplete, we made attempts to contact the original authors for further information [35, 36]. To reduce the risk of data entry errors, double checking was performed by CL. Any disparities were clarified by group discussion (CHL, CL and PHC).

### 2.5. Assessment of the risk of bias

The selected studies’ risk of bias was assessed by two reviewers (CHL and PHC) independently using the methodology and categories described in the Cochrane Collaboration Handbook [37]. Seven domains (sequence generation, allocation concealment, blinding of participate and personnel, blinding of outcome assessment, incomplete outcome data, selective reporting bias, other bias) were assigned a judgment of high, low or unclear risk. During disagreements, a group discussion was conducted to arrive at consensus [38]. Furthermore, we produced risk of bias graphs using the Review Manager 5.3 software [39].

### 2.6. Data synthesis and statistical analysis

To include all studies within one framework and prevent lower precision of estimating effect size, we chosed “lumping treatments” designs and made two assumptions that (1) we used the term no intervention to represent both observation and placebo (2) assumed different doses of the same drug were treated as the same treatment in the network.

#### 2.6.1 Dealing with missing data

We tried to contact the original authors to request relevant missing data and additional information. If the number of participants analyzed for a given outcome was not reported, the number of participants randomized per arm was used as the denominator. For dichotomous outcomes, numerators was used to calculate percentages if absolute number of events existed.

#### 2.6.2 Pairwise meta-analysis

We performed traditional pairwise meta-analyses for all comparisons for which there were head to head trials. For time to event outcomes (PFS and OS), treatment groups were compared using hazard ratios and their 95% CI; for dichotomous outcome (SAE), risk ratios was used. Both fixed-effects (FE) and random-effects (RE) meta-analysis was performed. Heterogeneity between studies was assessed by the Cochrane’s Q test and an I^2^ statistics. A p-value < 0.1 for the chi-square test was defined as statistically significant heterogeneity; an I^2^ statistics of 50% indicated high heterogeneity. We interpreted the result from the fixed-effects model if there was no significant heterogeneity, and random-effects models otherwise. Subgroup analyses were done in pairwise meta-analyses to explore causes of heterogeneity if heterogeneity exists; Subgroup analysis for the primary outcome by different maintenance therapy was performed; Statistical analysis was conducted by using R (meta package). The funnel plots were constructed to assess reporting biases.

#### 2.6.3 Network meta-analysis

To simultaneously compare all maintenance treatment options for CLL, an NMA was conducted based on the assumptions of homogeneity, similarity and consistency. The homogeneity assumption means the true treatment effects in a direct comparison of two treatments across studies are the same, which means there were no relevant heterogeneity between study results (which were assessed in pairwise comparisons). The similarity assumption means that each true treatment effect comparing any two treatments would be the same across all trials; or in other words, the trials do not differ in terms of any effect modifiers that would mean that treatment effects would not be expected to be the same across trials if all trials studied all treatments. The key assumption of similarity relates to potential clinical and methodological variation across the different comparisons. These differences may be reflected in the data in the form of disagreement in estimates between different source of evidence. Typically, consistency means statistically no difference between direct and various indirect effects that were estimated for the same comparison. We assessed the similarity/consistency assumption by comparing the characteristics of studies and also had planned to compare results from direct and indirect evidence by using a design-by-treatment interaction model method. In its simplest form, NMA is combination of direct and indirect estimates for relative treatment effects in one analysis [40]. The frequentist network meta-analysis approach was performed with R 3.4.2 software by using the “netmeta” package [41].

Under the framework of network meta-analysis, we ranked the evaluated regimens based on survival outcomes (OS and PFS) and safety (3–4 adverse events in each trial).

Hazard ratio with 95% confidence intervals (CI) were calculated using both the fixed-effect and random-effect frequentist NMA based on the UK NICE guidance [42]. We interpreted the fixed-effect NMA in the manuscript and described random-effect NMA at appendix. A network plot was produced to represent the overall information of the trials that were included for the analysis [40]. The contribution of each direct comparison to each network estimate was calculated according to the variance of the direct treatment effect and the influence of the network structure.[43].

We used surface under the cumulative ranking (SUCRA) to rank the intervention’s hierarchy in the network meta-analysis and then we estimated mean ranks. [44–46]. The larger the SUCRA value (ie, closer to 1) is, the better the rank of the intervention [41, 47]. Forest plots present the results of NMA and summary relative mean effects, 95% confidence intervals and SUCRA for all comparisons together [48]. To evaluate balance of benefit and harm of interventions, we constructed scatter plot to compare the SUCRA value of progression free survival and serious adverse events using STATA software (Version 13.0; Stata Corporation, College Station, Texas, USA).

We evaluated whether treatment effects for the primary outcome were robust by sensitivity and subgroup analysis with fixed-effect pairwise meta-analysis and network meta-analysis. Variables included blinding (double blind, open label), comparison (placebo, observation), time to maintenance therapy (post-front line therapy, post-secondary or later-line therapy) and trial with early termination. To examine the key assumptions of NMA, clinical comparisons of basic characteristics of included trials and statistic network meta-regression model was administered. Characteristics of population demography (mean age, complete remission percentage before maintenance therapy, percentage of mutated IGHV), median follow up duration, study quality, PFS and OS were compared. We also intended to conduct network meta-regression to help identify effect modifiers and assess the key assumptions of NMA, however, it was difficult to conduct because of the limited number of studies. Comparison-adjusted-funnel plot was also conducted to assess publication bias of NMA.

The review protocol was registered at International Prospective Register of Systematic Reviews (PROSPERO, CRD42018109514).

## 3. Results

### 3.1. Basic characteristics

**Fig 1:**
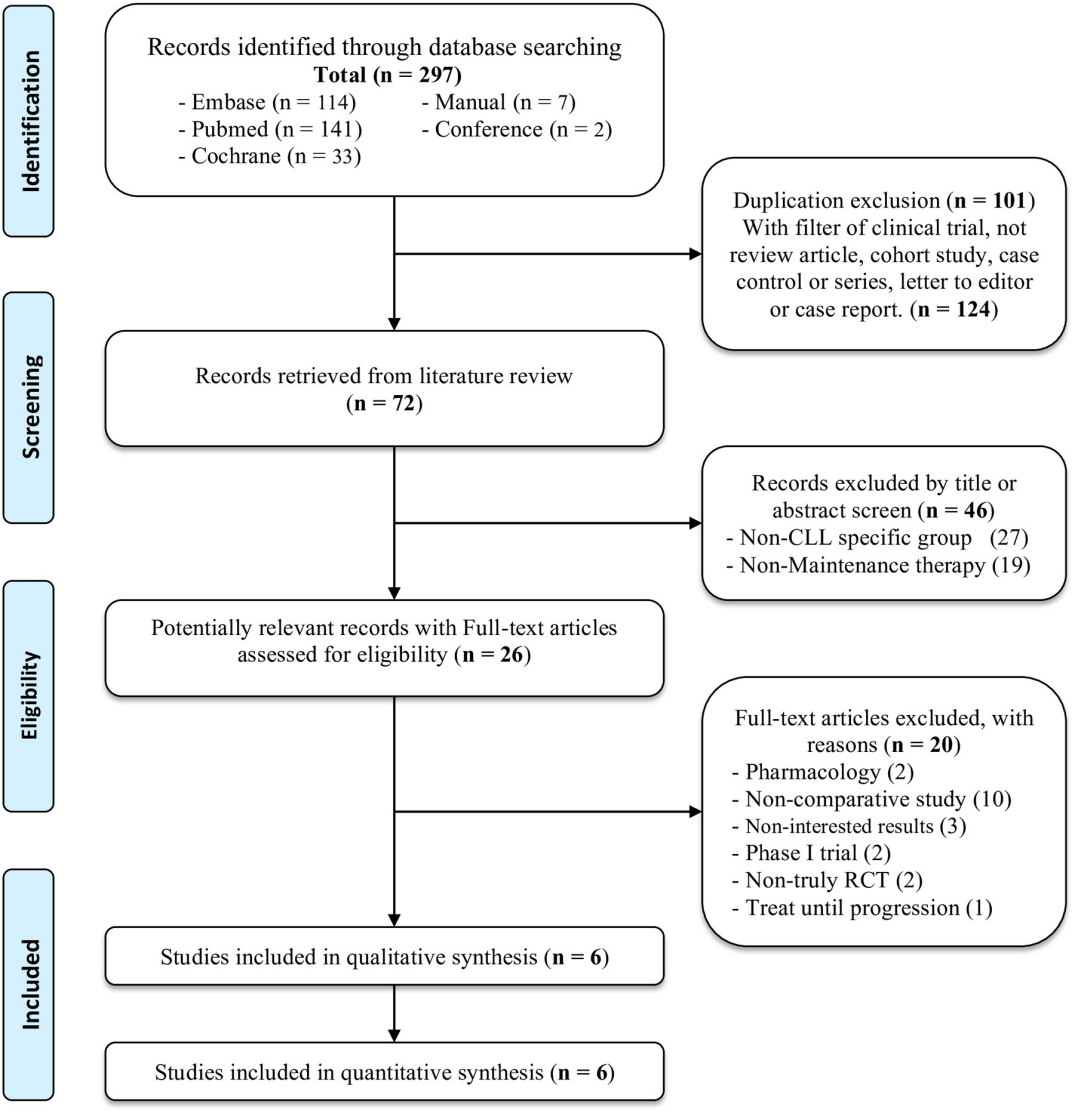
PRISMA flowchart of network meta-analysis.

Fig 1 shows search algorithms used for the present study. A total of 297 citations were searched. After removal of duplication and non-RCTs, 72 studies were selected for screening of titles/abstracts, of which 26 potentially relevant records were assessed for eligibility and six RCTs were then included in the analysis (S3 Appendix).

Table 1 provides the basic characteristics of the studies that were included, all of which were high-quality phase III RCTs (S1 Fig). All six trials presented low risk in selection and reporting bias domains. In terms of performance and detection bias, two double-blind design studies with low risk in blinding and domains focused on lenalidomide [25, 26]; three open-label design studies focused on rituximab [27, 28, 49]; and one open-label design study focused on ofatumumab [50]. PALG CLL4 study had high risk of attrition bias due to premature termination [49]. All included trials were analyzed with intention-to-treat design. No obvious selective outcome reporting bias was found.

**Table 1:** Basic characteristics of included randomized trials.

Trial Name Registration Code Year	Study Design	Treatment Design		Cases	Mean Age	Follow up/Analysis	Front-line cases(%)/FCR regimen(%)	CR/PR(%)	Front-line regimens
CONTINUUM (NCT00774345) 2017	Phase III, DB, MC, RCT	Lenalidomide v.s. Placebo	Oral 2.5 mg/day (if tolerated, maximal escalated to 5 mg/day)	160 v.s. 154	63.0 v.s. 63.0	31.5 months (18.9–50.8) ITT	28%/98.9%	23.90%/76.10%	FCR, chlorambucil, alemtuzumab
CLLM1 (NCT01556776) 2017	Phase III, DB, MC, RCT	Lenalidomide v.s. Placebo	Oral 5 mg/day (if tolerated, maximal escalated to 15 mg/day)	60 v.s. 29	64.0 v.s. 64.0	17.9 months (9.1–28.1) ITT	100%/22.1%	39.30%/60.70%	FCRB
CLL 2007 SA (NCT00645606) 2017	Phase III, OP, MC, RCT	Rituximab v.s. Observation	Intravenous 500 mg/m^2^ 2-monthly intervals for 2 years (maximum of 12 cycles)	202 v.s. 207	71.7 v.s. 71.1	47.9 months (32.4–65.1) ITT	100%/100%	36.7%/73.30%	FCR
AGMT CLL-8a (NCT01118234) 2016	Phase III, OP, MC, RCT	Rituximab v.s. Observation	Intravenous 375 mg/m^2^ 3-monthly intervals for 2 years	134 v.s. 129	63.0 v.s. 63.0	33.4 months (25.7–42.8) ITT	79.8%/91.9%	56.3%/43.7%	FCRB
PALG-CLL4 (NA) 2018	Phase III, OP, MC, RCT	Rituximab v.s. Observation	Intravenous 375 mg/m^2^ 3-monthly intervals for 2 years	33 v.s. 33	57.8 v.s. 57.3	NA (Prematurely terminated) ITT	100%/0%	22.7%/50.5%	RCC
PROLONG (NCT00802737) 2015	Phase III, OP, MC, RCT	Ofatumumab v.s. Observation	Intravenous first dose of 300 mg, increased to 1000mg next week, then 2-monthly intervals for 2 years	238 v.s. 236	64.0 v.s. 65.0	19.1 months (10.3–28.8) ITT	Only enrolled received second-line/42.8%	19.20%/80.80%	FCR, FR, BR, R-CVP, Alkylating monotherapy

**CR**: Complete response, **PR**: Partial response, **OP**: Open label; **DB**: double blind, **MC**: Multiple centers, **RCT**: randomized controlled trial, **ITT**: intention-to-treat,

**RCC**: Rituximab, Cladribine and Cyclophosphamide, **FCR**: Fludarabine, Cyclophosphamide and Rituximab, **FR**: Fludarabine and Rituximab, **BR**: Bendamustine and Rituximab, **R-CVP**: Bendamustine, Cyclophosphamide, Vincristine and Predisone, **FCRB: F**ludarabine, Cyclophosphamide, Rituximab and Bendamustine

The mean age ranged from 57.3 to 71.7 years, whereas the median follow-up duration ranged from 17.9 to 47.9 months. Three trials enrolled patients who had received only first-line therapy [25, 27, 49], one trial enrolled patients who received at least two-lines of therapy [50], and the remaining two trials were mixed [26, 28]. Among three rituximab maintenance trials, CLL 2007 SA trial [27] recruited patients in responses to completed FCR induction therapy; AGMT CLL-8a trial [28] included patients who had response to first-line or second-line rituximab-containing chemoimmunotherapy; PALG-CLL4 trial [49] focused on patients with rituximab, cladribine, and cyclophosphamide (RCC) induction and received subsequent maintenance with rituximab. The remission response to previous treatments varied from trials. A complete response ranged from 19.2% to 56.3%, whereas a partial response ranged from 43.7% to 80.8%.

### 3.2. Pair-wise meta-analysis

**Fig 2:**
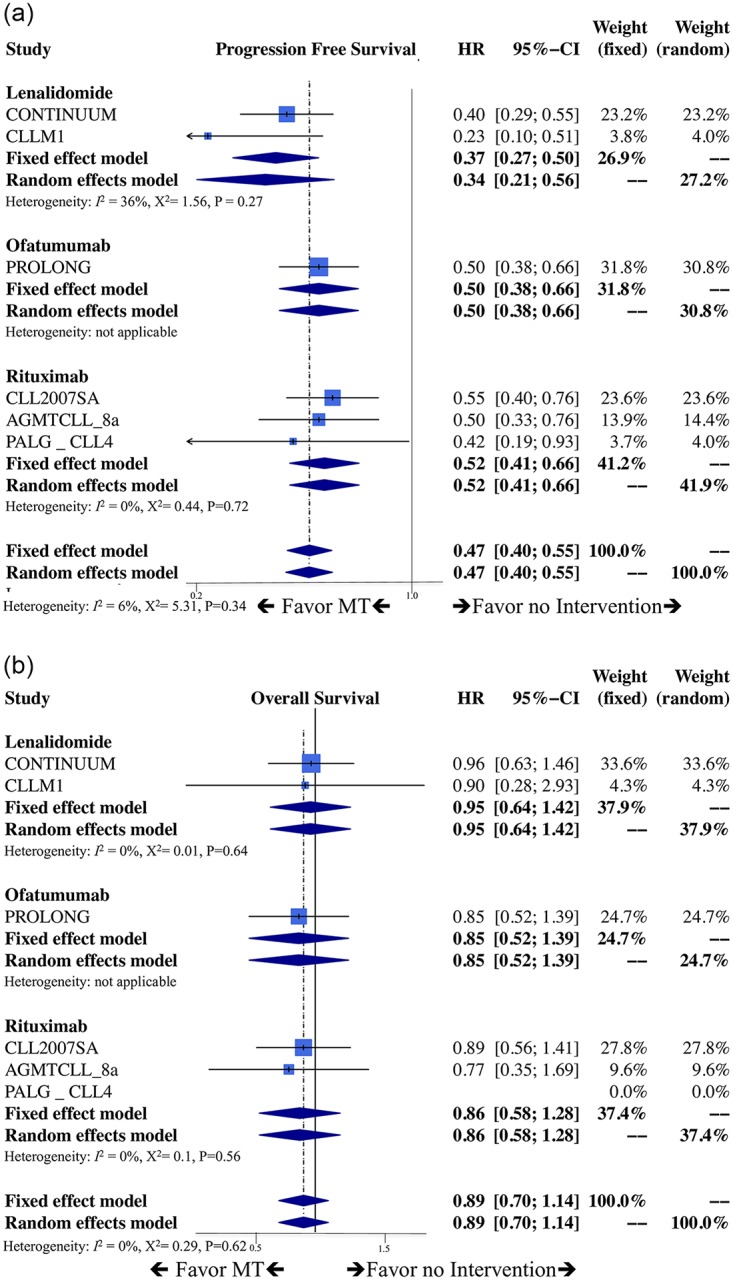
Pair-wise meta-analysis. **a. Forest plot of progression-free survival**. HR: hazard ratio, CI: confidence interval, MT: maintenance therapy, OBS: no intervention. **b. Forest plot of overall survival**. HR: hazard ratio, CI: confidence interval, MT: maintenance therapy, OBS: no intervention.

For the PFS result of pairwise meta-analysis, six trials with total of 1,615 patients were included [25–28, 49, 50], Fig 2 shows statistically significant efficacy of maintenance therapy for reduction risk of disease progression when compared with no intervention (HR: 0.47, 95% CI: 0.40–0.55, P<0.05, I^2^:6%, Cochrane Q: 5.31; fixed-effect model, Fig 2A). Lenalidomide had superior effect versus no intervention (HR:0.34, 95%CI:0.21–0.56, I^2^:36%); Rituximab had superior effect versus no intervention (HR:0.52, 95%CI:0.41–0.66, I^2^:0%); Ofatumumab had superior effect versus no intervention (HR:0.50, 95%CI:0.38–0.66, I^2^:N/A).

In terms of OS, the effect of maintenance therapy showed no significant difference versus no intervention (HR: 0.89, 95% CI: 0.70–1.14, P = 0.89, I^2^:0%, Cochrane Q:0.29: fixed-effect model, Fig 2B). Each maintenance therapy versus no intervention individually also had no significant difference in OS (Lenalidomide with HR:0.95, 95%CI:0.64–1.42, I^2^:0%; Rituximab with HR:0.86, 95%CI:0.58–1.28, I^2^:0%; Ofatumumab with HR:0.85, 95%CI:0.52–1.39, I^2^:N/A).

Furthermore, maintenance therapy showed significant increased incidence of SAE versus no intervention (RR: 1.54, 95% CI: 1.19–1.99, P = <0.05, I^2^:88%, Cochrane Q:42.34; random-effect model, S2 Fig). There were no significant publication bias detected among the studies according to the funnel plot and Egger’s test (p>0.05) (S2 Fig).

### 3.3. Network meta-analysis

**Fig 3:**
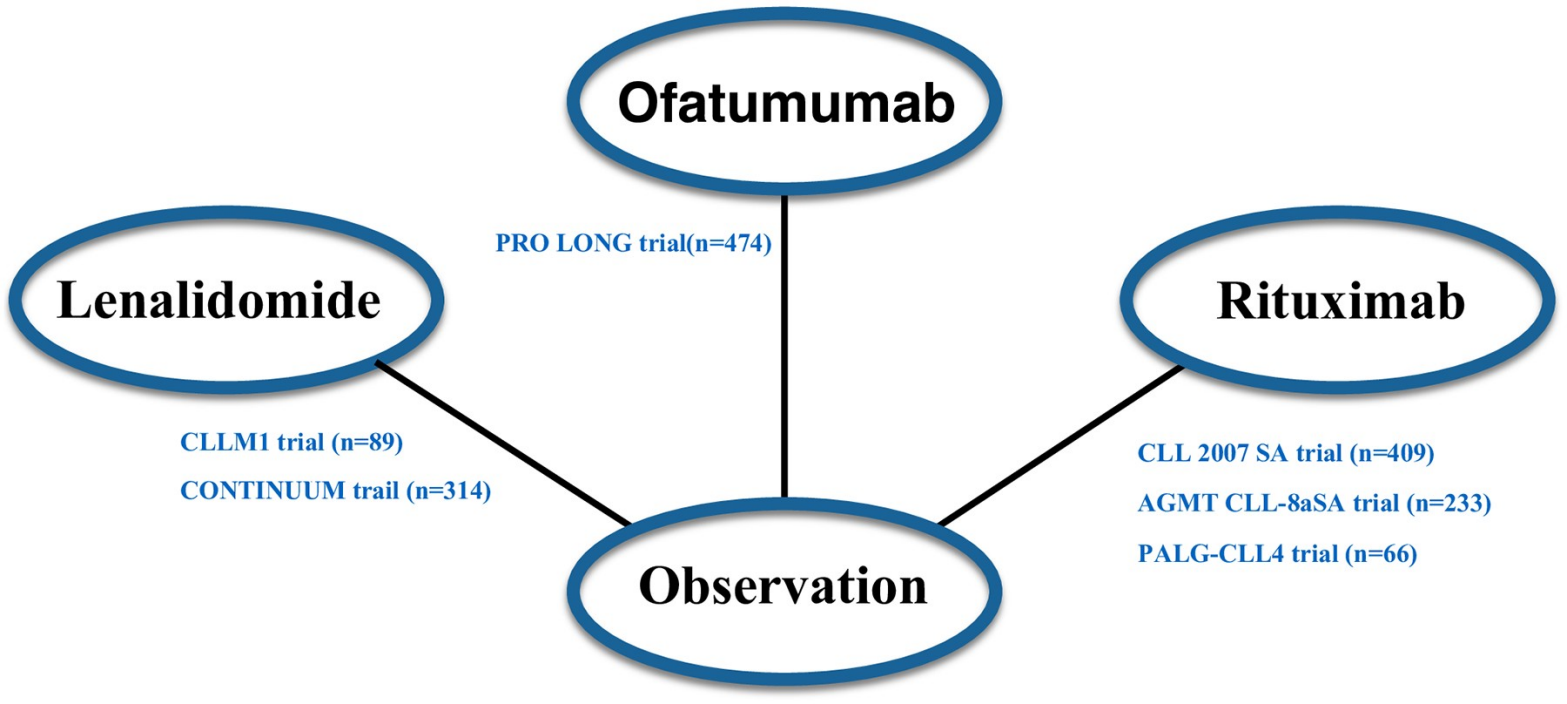
A schematic of the network of evidence used in the network meta-analysis. Directly comparable treatments are linked with a line; n: number.

Fig 3 shows the schematic network of eligible comparisons for maintenance therapy in CLL patients. We compared basic characteristics of included trials to examine the key assumptions of NMA. We looked at the following variables mean age, complete remission rates, IgHV mutation percentage, time of follow up, and the methodologic quality of the study [27] and found no statistically significant differences (S3 Fig) Note that there were no head-to-head trials that compared different maintenance therapies; therefore, the synthesized results between interventions were produced either from qualified indirect or direct evidence but not both. The connections within our network are sparse and the treatment comparisons were therefore estimated by either direct or indirect evidence but not both. Consequently, it is not feasible to evaluate the inconsistency in treatment effects between direct and indirect comparisons.

#### 3.3.1 Clinical efficacy (progression free survival & overall Survival)

**Fig 4:**
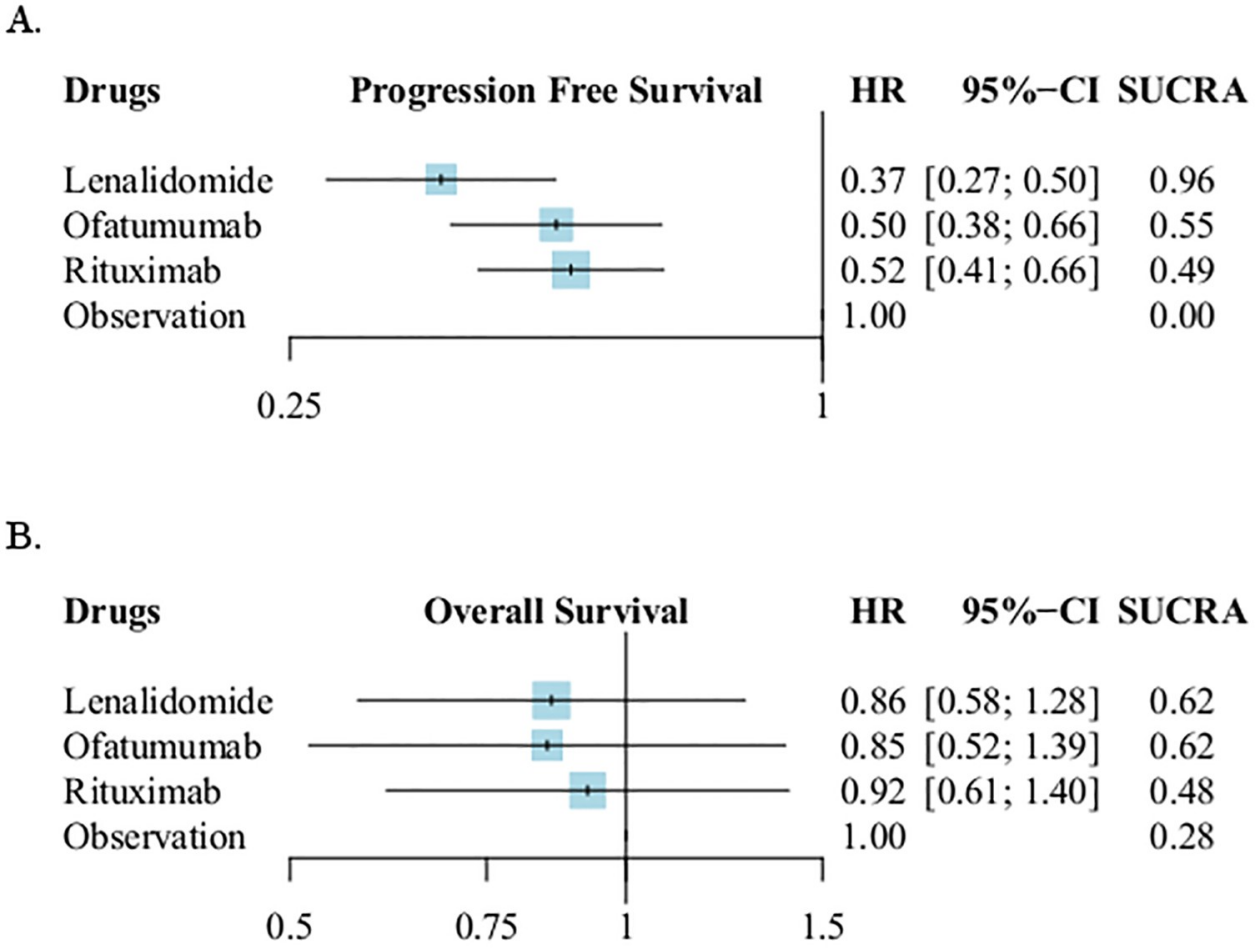
Network meta-analyses results of progression free survival and overall survival. **a. Forest plot of progression-free survival**. NMA, network meta-analysis; HR: hazard ratio, CI: confidence interval, SUCRA: surface under the cumulative ranking curve. **b. Forest plot of overall survival**. NMA, network meta-analysis; HR: hazard ratio, CI: confidence interval, SUCRA: surface under the cumulative ranking curve.

A total of six trials [25–28, 49, 50] that contained 1,615 patients were included for PFS (Fig 4A) and OS (Fig 4B) analyzes. “No intervention” was set as reference comparator. According to the NMA results, in terms of prolongation of progression-free survival, treatment with lenalidomide demonstrated the highest relative efficacy for prolonging PFS (HR: 0.37, 95% CI: 0.27–0.50, SUCRA:0.96); ofatumumab and rituximab showed similar effect to reduce the risk of disease progression with HR 0.50 (95% CI:0.38–0.66, SUCRA:0.55) and 0.52 (95% CI:0.41–0.66, SUCRA:0.49), respectively. Among the active maintenance therapies, the NMA demonstrated HR 0.71 (95%CI: 0.49–1.05, P = 0.08) when lenalidomide compared to rituximab; HR 0.741 (95%CI: 0.49–1.11) when lenalidomide compared to ofatumumab; HR 0.96 (95%CI: 0.67–1.39) when ofatumumab compared to rituximab (S4 Fig).

In terms of OS, the network meta-analysis reported lenalidomide, rituximab and ofatumumab had no statistically significant differences in prolongation of OS compared with no intervention (Fig 4B). The detailed NMA results of OS and PFS were presented in Table 2. The contribution plots and comparison-adjusted funnel plots were presented in S4 Fig and there were no significant publication bias existed.

**Table 2:** Overall network meta-analysis results of progression free survival and overall survival.

**Lenalidomide**	1.122 (0.597 to 2.109)	1.112 (0.633 to 1.952)	0.954 (0.641 to 1.418)
0.741 (0.494 to 1.111)	**Ofatumumab**	0.991 (0.526 to 1.867)	0.851 (0.521 to 1.391)
0.713 (0.486 to 1.046)	0.962 (0.668 to 1.386)	**Rituximab**	0.862 (0.584 to 1.280)
**0.370 (0.275 to 0.499)**	**0.501 (0.381 to 0.662)**	**0.522 (0.412 to 0.660)**	**Observation**

The bottom half shows the results of network meta-analyses of progression free survival (hazard ratio and 95% confidence interval);

The top half shows the results of overall survival (hazard ratio and 95% confidence interval). Boldface letter indicates statistical significance.

#### 3.3.2 Clinical safety (serious adverse events)

**Fig 5:**
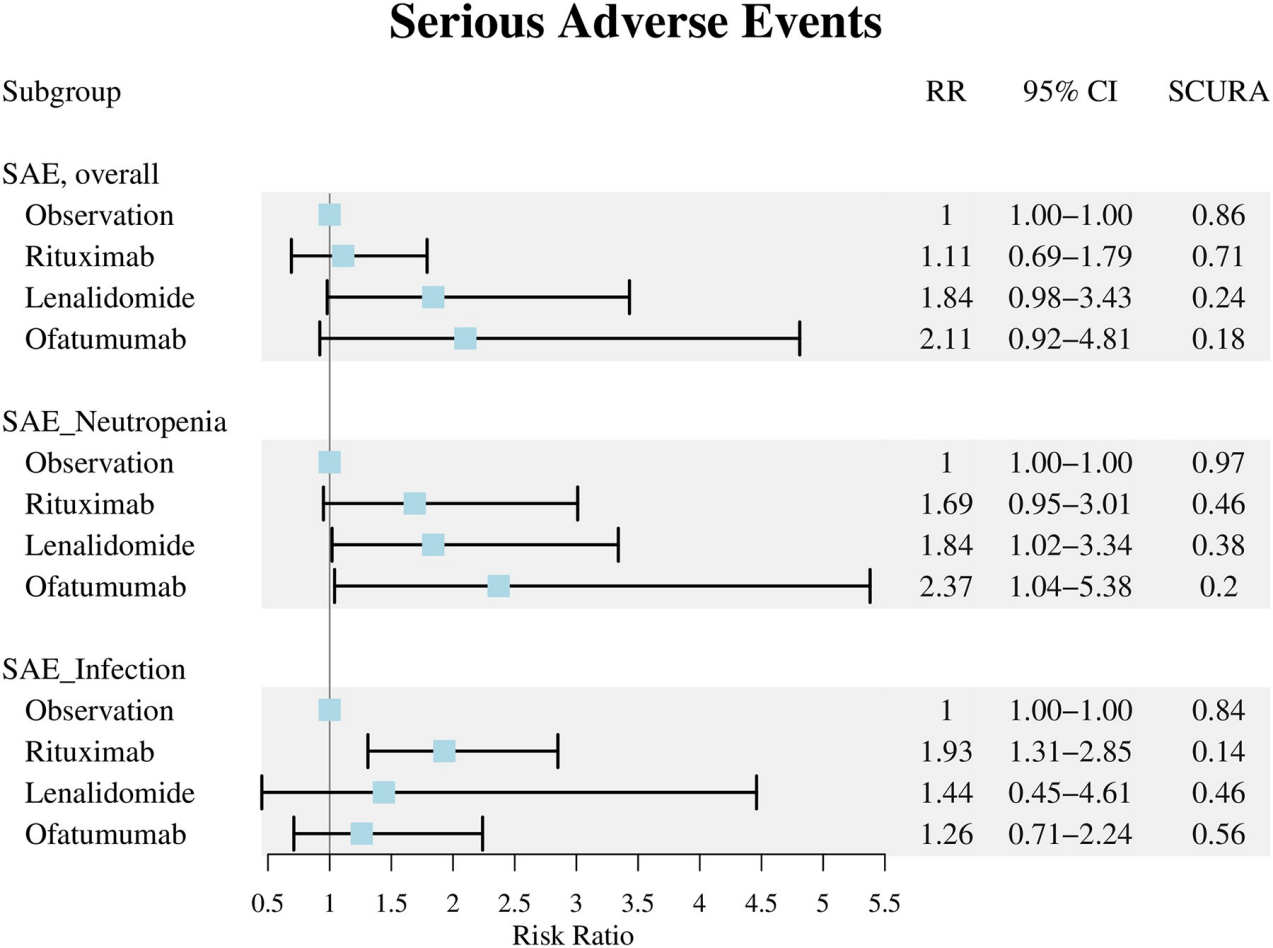
Forest plot of serious adverse events. We used number of patients randomised in CLLM1 trial due to prematurely termination of the study. RR: risk ratio, CI: confidence interval, SUCRA: surface under the cumulative ranking curve, SAE: serious adverse events.

In terms of safety, detailed results of pooled analysis are described in Fig 5. Compared with no intervention, lenalidomide, rituximab, and ofatumumab exhibited no significant difference in increasing incidence of overall SAE (RR:1.84, 95% CI:0.98–3.43 of lenalidomide; RR:1.11, 95% CI:0.69–1.79 of rituximab; RR:2.11, 95% CI:0.92–4.81 of ofatumumab, separately) [25–28, 49, 50]. However, lenalidomide and ofatumumab had statistically significant increased the risk of serious neutropenia events by approximately two-fold (RR: 1.84, 95% CI:1.02–3.34 of lenalidomide; RR:2.37, 95% CI:1.04–5.38 of ofatumumab) versus no intervention [25–28, 50]. Although rituximab showed neutral safety in terms of overall SAE and serious neutropenia events, the NMA showed rituximab had statistically significant increasing the risk of serious infectious events (RR: 1.93, 95% CI: 1.31–2.85) versus no intervention [25–28, 50].

#### 3.3.3 Sensitivity analyses, subgroup analyses and scatter plots of benefit and harms

To assess the potential bias, sensitivity analyses were conducted by the study design (excluding double-blind studies) and the quality assessments (excluding low quality studies). The result did not alter through the sensitivity analyses. Furthermore, details of subgroup analysis results were described in S5 Fig.

**Fig 6:**
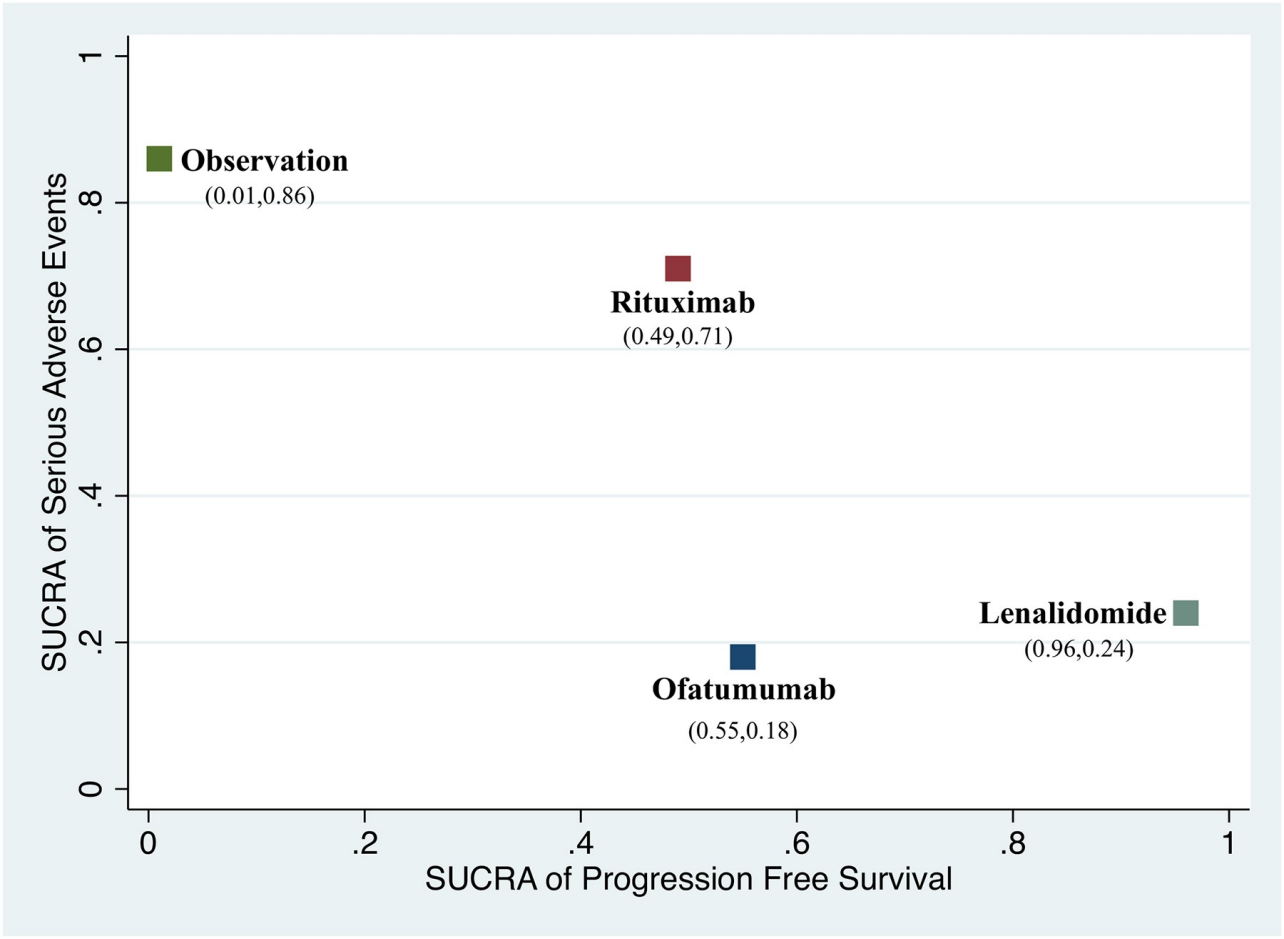
Scatter plot presenting the ranking of progression-free survival and serious adverse events. The more close to right top half means better prolongation of PFS and lower incidence of SAE. X-axis represents SUCRA value of PFS; Y-axis presents SUCRA value of serious adverse events. Both of the values were showed in brackets.

Fig 6 illustrating the SUCRA value of PFS and SAE for all comparisons. Among the maintenance therapies, lenalidomide showed best favorable efficacy of PFS but with higher risk of SAE versus rituximab.

## 4. Discussion

To our best knowledge this is the first network meta-analysis to compare relative efficacy and safety of all current available maintenance therapies for CLL patients. The results demonstrated that all maintenance therapies achieved superior effect in prolongation of PFS compared with no intervention. In the same time, administration of maintenance therapy did not significantly increase the risk of overall SAE compared with no intervention. Lenalidomide exhibited the most favorable efficacy in reduction risk of disease progression within maintenance therapy with the highest probability of being the best treatment in prolongation of PFS (SUCRA: 96%).

However, unlike in PFS, there is no evidence that maintenance therapy options prolong OS currently. A possible reason for this may be that CLL is a hematological disease that progresses slowly. Because of the limited follow-up duration in published clinical trials, only the PROLONG trial reached the median OS. In slowly progressive disease with long OS and availability of effective treatments, OS may turn out to be an infeasible endpoint to test due to crossover effects and the required long-term follow up duration. In the current era, the treatment options change much quicker than even the most optimistic turn-around times for OS results, OS becomes a highly impractical measurement. Thus, surrogates have been developed to more quickly assess the impact of a therapy for such disease. In a meta-analysis, progression free survival (PFS) has been shown to be a surrogate for OS in the era of chemotherapy and chemoimmunotherapy [51]. There were much discussions about the strengths and weaknesses of considering PFS as a surrogate endpoint for OS [52–54]. The key issue is how to validate the association between PFS and OS. However, based on current best evidence, OS will not be an appropriate primary outcome until more long-term follow-up trials emerged. Maintenance therapy did not increase risk of overall SAE compared with no intervention. Among classifications of SAE, hematological and infectious adverse events were the most commonly observed events; thus, NMA confirmed that lenalidomide and ofatumumab had significantly increased risk of serious neutropenia events. The CONTINUUM [26] and CLL 2007 SA [27] trials reported relatively higher incidence rates of grade 3–4 neutropenia (60% and 53%, respectively). Thus, theoretically, the higher incidence of neutropenia could lead to a higher occurrence of infectious events in patients with CLL. However, even with >50% incidence of serious neutropenia, the incidence of infections were <20%, and the highest incidence of infections was found only in the CLL 2007 SA trial [27]. Moreover, there were few fatal adverse events in the CONTINUUM trial investigating maintenance therapy using lenalidomide. Note that three deaths were reported in that trial, which were associated with infection and neutropenia. Thus, treating physicians should closely monitor the occurrence of hematological adverse events and infectious events in patients with CLL undergoing maintenance therapy.

The current guidelines of the NCCN [13] recommend that first-line lenalidomide consolidation therapy should be administered after chemoimmunotherapy for patients with high-risk unmutated IgHV or del(17p)/TP53 mutation. Furthermore, the selection of agents for second-line maintenance therapy is lenalidomide or ofatumumab determined by the treating physician; however, the assessment/detection of poor prognosis factors, such as del(17p)/TP53 mutations, IgHV unmutated configuration, and short telomeres is not available in all hospitals. Thus, the synthetic NMA approach could provide evidence, assisting physicians for clinical decision-making.

The currently available trials of maintenance therapy were conducted during the chemotherapy-based era. In the last five years, the use of small molecular kinase inhibitors (SMKIs) has demonstrated promising results for treating CLL, regardless of first- or second-line treatment. Therefore, the role of maintenance therapy in the era of targeted therapy is doubtful. In high-risk CLL patients, traditional chemoimmunotherapy combined with maintenance therapy may have a role in maintaining response of innovative SMKIs treatment or failing to the novel combination treatment. Moreover, a recent study involving CLL patients treated with Bruton’s tyrosine kinase inhibitor, ibrutinib, showed that patients possibly evolved into the Richter transformation, which is defined as a transformation of CLL into a more aggressive type of lymphoma (i.e., diffuse large B-cell lymphoma) [55]. However, in the PROLONG trial, none of the CLL patients treated with ofatumumab maintenance therapy developed the Richter transformation; thus, additional large clinical trials are required. Despite the demonstrated effectiveness of SMKIs-based therapy, the economic burden associated with this approach requires the use of conventional chemotherapy-based therapy for current clinical practice.

Note that our study has four limitations. First, the six selected studies were all maintenance therapy compared with no intervention rather than head-to-head comparisons between maintenance therapies. Even the included trials fitted the homogeneity and similarity assumptions of NMA, the assessment of the consistency assumption was not possible because both direct and indirect evidence did not contribute to any single treatment comparison. Second, the details of maintenance therapy had a little different, the different dosage or treatment intervals existed in different trials. lenalidomide had a variable initial dosage (2.5–5.0 mg) and was titrated to a maximal dosage of 5–15 mg; moreover, rituximab had a variable dosage and treatment intervals (375 mg/m^2^ with 3-month intervals and 500 mg/m^2^ with 2-month intervals). Third, the previous treatment regimens were different and the FCR regimen was the most frequently used first-line regimen. Finally, basic treatment responses of patients to previous therapies were different with complete response ranging from 19.2% to 56.3%.

## 5. Conclusions

Based on the present evidence, maintenance therapy may considered being administered in CLL patients who have achieved at least partial response following previous therapy. Lenalidomide, rituximab, and ofatumumab prolong PFS, significantly reducing the risk of disease progression compared with no intervention. Lenalidomide demonstrated the highest efficacy in prolongation of PFS, whereas all maintenance therapies showed similar benefit in OS. In the absence of direct head-to-head comparison trials to compare different maintenance therapies, NMA could provide useful estimates for the relative efficacy and safety of maintenance therapies in CLL.

## Supporting information

S1 Appendix Search strategies and detailed records. (DOCX)

S2 Appendix PRISMA NMA checklist. (DOCX)

S3 Appendix Exclusion of papers after full-text screening. (DOCX)

S1 Fig Quality assessment (Cochrane risk of bias). (DOCX)

S2 Fig Detail results of pairwise meta-analyses. (DOCX)

S3 Fig Tabulated summary of average characteristics. (DOCX)

S4 Fig Detail results of network meta analyses. (DOCX)

S5 Fig Subgroup analysis of primary outcome (PFS). (DOCX)

S6 Fig Network meta-analysis with random-effect model. (DOCX)

S1 Data (XLSX)

## Abbreviations

CI: confidence interval
CLL: chronic lymphocytic leukemia
FCR: fludarabine, cyclophosphamide, and rituximab
HR: hazard ratio
IgHV: immunoglobulin heavy chain (IgVH) mutational status
NMA: network meta-analysis
OR: odds ratio
OS: overall survival
PFS: progression free survival
RCT: randomized controlled trials
RR: relative risk
SMKIs: small molecular kinase inhibitors
SUCRA: surface under cumulative ranking curve
