# Supplementary material for: A network meta-analysis of maintenance therapy in chronic lymphocytic leukemia
Source: PLoS One. 2020 Jan 29;15(1):e0226879. doi: 10.1371/journal.pone.0226879 (PMC6988939; doi:10.1371/journal.pone.0226879)
Supplement: S1 Appendix — (DOCX) [file pone.0226879.s001.docx]

**Supplementary appendix**

**A Network Meta-analysis of Maintenance Therapy in Chronic Lymphocytic Leukemia**

Cho-Hao Lee, Po-Huang Chen, Chin Lin, Chieh-Yung Wang, Ching-Liang Ho

Contents

Supplementary Appendix 1. Search strategy

Supplementary Appendix 2. PRISMA checklist

Supplementary Appendix 3. Included full-text screening studies and on-going trials

Supplementary Figures 1. Risk of bias

Supplementary Figures 2. Detail results of Pairwise meta-analyses

Supplementary Figures 3. Tabulated summary of average characteristics

Supplementary Figures 4. Detail results of Network Meta-analyses

Supplementary Figures 5. Subgroup and Sensitivity Analysis of primary outcome (PFS)

Supplementary Figures 6. Network meta-analysis with random-effect model

| **Relevant text of Population & Type**   1. B cell Leukemia 2. Lymphocytic leukemia 3. Chronic lymphocytic leukemia 4. Small lymphocytic leukemia   A= #1 or #2 or #3 or #4   1. Maintenance 2. Maintenance therapy 3. Consolidation 4. Consolidation therapy   B = #5 or #6 or #7 or #8   1. Trial 2. Clinical trial/ trials 3. Randomized 4. Randomization 5. Controlled trial/trials 6. Randomized controlled trial/trials 7. Controlled clinical trial 8. RCT 9. Persepctive study 10. Clinical study 11. Clinical article   C = #9 or #10 or #11 or #12 or #13 or #14 or #15 or #16 or #17 or #18 or #19   1. Progression free survival 2. Overall Survival 3. Response 4. Adverse Events 5. Mortality   D = #20 or #21 or #22 or #23 or #24 | **Relevant text of Intervention & Outcome**   1. Anti-CD 20 2. Rituximab 3. Mabthera 4. Rituxan 5. Lenalidomide 6. Revlimidr 7. HuMax-CD20 8. Ofatumumab 9. Arzerra 10. Bruton’s tyrosine kinase inhibitor 11. Ibrutinib 12. Imbruvica 13. Phosphoinositide 3 kinase **δ** inhibitor 14. Idelalisib 15. Zydelig 16. Venetoclax 17. Venclexta 18. Obinutuzumab 19. Gazyva   E = #1 or #2 or #3 or #4 or #5 or #6 or #7 or #8 or #9 or #10 or #11 or #12 or #13 or #14 or #15 or #16 or #17 or #18 or #19  *. All search keyword with [Mesh Terms] or [All Fields]  **PUBMED**: <http://www.ncbi.nlm.nih.gov/pubmed>  **EMBASE**:  https://www.embase.com  **COCHRANE CENTRAL**: <https://www.cochrane.com> |
| --- | --- |

**Appendix 1**: Search strategies and detailed records
