# Supplementary material for: A network meta-analysis of maintenance therapy in chronic lymphocytic leukemia
Source: PLoS One. 2020 Jan 29;15(1):e0226879. doi: 10.1371/journal.pone.0226879 (PMC6988939; doi:10.1371/journal.pone.0226879)
Supplement: S3 Appendix — (DOCX) [file pone.0226879.s003.docx]

**Appendix 3**: Exclusion of papers after full-text screening

| Autohrs/  Publish Year | Title | *Journal* | Comment |
| --- | --- | --- | --- |
| Weiss, M. A.  2000 | Consolidation therapy with high-dose cyclophosphamide improves the quality of response in patients with chronic lymphocytic leukemia treated with fludarabine as induction therapy | *Leukemia, 14*(9), 1577-1582. | Non-comparative  study |
| Byrd, J. C.  2003 | Randomized phase 2 study of fludarabine with concurrent versus sequential treatment with rituximab in symptomatic, untreated patients with B-cell chronic lymphocytic leukemia: results from Cancer and Leukemia Group B 9712 (CALGB 9712) | *Blood, 101*(1), 6-14. | Compared concurrent versus sequential |
| Hainsworth, J. D  2003 | Single-agent rituximab as first-line and maintenance treatment for patients with chronic lymphocytic leukemia or small lymphocytic lymphoma: a phase II trial of the Minnie Pearl Cancer Research Network. | *Clin Oncol, 21*(9), 1746-1751 | Non-comparative  study |
| Thieblemont, C.  2004 | Maintenance therapy with a monthly injection of alemtuzumab prolongs response duration in patients with refractory B-cell chronic lymphocytic leukemia/small lymphocytic lymphoma (B-CLL/SLL) | *Leuk Lymphoma, 45*(4), 711-714 | Non-comparative  study |
| Del Poeta, G.  2008 | Consolidation and maintenance immunotherapy with rituximab improve clinical outcome in patients with B-cell chronic lymphocytic leukemia. | *Cancer, 112*(1), 119-128. | Non-comparative  study |
| Hainsworth, J. D.  2008 | Combination therapy with fludarabine and rituximab followed by alemtuzumab in the first-line treatment of patients with chronic lymphocytic leukemia or small lymphocytic lymphoma: a phase 2 trial of the Minnie Pearl Cancer Research Network | *Cancer, 112*(6), 1288-1295. | Non-comparative  study |
| Lamanna, N., & Weiss, M. A  2008 | Consolidation and maintenance rituximab therapy in chronic lymphocytic leukemia | *Curr Oncol Rep, 10*(5), 363-364. | Non-RCT |
| Bosch, F.  2009 | Rituximab, fludarabine, cyclophosphamide, and mitoxantrone: a new, highly active chemoimmunotherapy regimen for chronic lymphocytic leukemia | *J Clin Oncol, 27*(27), 4578-4584. | Non-comparative  study |
| Lin, T. S.  2010 | Consolidation therapy with subcutaneous alemtuzumab after fludarabine and rituximab induction therapy for previously untreated chronic lymphocytic leukemia: final analysis of CALGB 10101 | *J Clin Oncol, 28*(29), 4500-4506 | Non-comparative  study |
| Kaufman, M. S.  2011 | Alemtuzumab maintenance may safely prolong chemotherapy-free intervals in chronic lymphocytic leukemia. | *Med Oncol, 28*(2), 532-538. | Non-  comparative  study |
| Sehn, L. H  2012 | A phase 1 study of obinutuzumab induction followed by 2 years of maintenance in patients with relapsed CD20-positive B-cell malignancies. | *Blood, 119*(22), 5118-5125 | Phase I trial |
| Abrisqueta, P.  2013 | Rituximab maintenance after first-line therapy with rituximab, fludarabine, cyclophosphamide, and mitoxantrone (R-FCM) for chronic lymphocytic leukemia. | *Blood, 122*(24), 3951-3959 | Non-comparative  study |
| Shanafelt, T. D.  2013 | Long-term repair of T-cell synapse activity in a phase II trial of chemoimmunotherapy followed by lenalidomide consolidation in previously untreated chronic lymphocytic leukemia (CLL) | *Blood, 121*(20), 4137-4141 | pharmacology study |
| Foa, R.  2014 | Chlorambucil plus rituximab with or without maintenance rituximab as first-line treatment for elderly chronic lymphocytic leukemia patients | *Am J Hematol, 89*(5), 480-486 | No interested data (Hazard ratio of PFS & OS data were not available) |
| Huang, B. T.  2014 | How to determine post-FCR therapy for cytogenetic risk-tailored elderly patients with chronic lymphocytic leukemia, maintenance rituximab or observation | *Med Oncol, 31*(8), 104 | open-label, prospective, observational study |
| Maddocks, K.  2014 | A dose escalation feasibility study of lenalidomide for treatment of symptomatic, relapsed chronic lymphocytic leukemia | *Leuk Res, 38*(9), 1025-1029 | pharmacology study |
| Mato, A. R.  2015 | Reduced-dose fludarabine, cyclophosphamide, and rituximab (FCR-Lite) plus lenalidomide, followed by lenalidomide consolidation/maintenance, in previously untreated chronic lymphocytic leukemia | *Am J Hematol, 90*(6), 487-492 | Non-comparative  study |
| Awan, F. T.  2016 | A phase 1 clinical trial of flavopiridol consolidation in chronic lymphocytic leukemia patients following chemoimmunotherapy. | *Ann Hematol, 95*(7), 1137-1143 | Phase 1 trial |
| Flinn, I. W.  2016 | A phase II study of two dose levels of ofatumumab induction followed by maintenance therapy in symptomatic, previously untreated chronic lymphocytic leukemia. | *Am J Hematol, 91*(10), 1020-1025. | Phase II study with cohort control |
| Strati, P.  2017 | Consolidation treatment with lenalidomide following front-line or salvage chemoimmunotherapy in chronic lymphocytic leukemia. | *Haematologica, 102*(12), e494-e496 | No interested data |
| Cramer, P.  2018 | CLL2-BXX Phase II trials: sequential, targeted treatment for eradication of minimal residual disease in chronic lymphocytic leukemia. | *Future Oncol, 14*(6), 499-513 | kinase inhibitors trials with treatment until progression design |

Other maintenance therapies with no recommendation available for current practice

| Autohrs/  Publish Year | Title | *Journal* | Comment |
| --- | --- | --- | --- |
| Ferrara, F.  1992 | Recombinant interferon-alpha 2A as maintenance treatment for patients with advanced stage chronic lymphocytic leukemia responding to chemotherapy. | *Am J Hematol, 41*(1), 45-49 | Interferon |
| Zinzani, P. L  1994 | Alpha-interferon as maintenance drug after initial fludarabine therapy for patients with chronic lymphocytic leukemia and low-grade non-Hodgkin's lymphoma | *Haematologica, 79*(1), 55-60 | Interferon |
| O'Brien, S.  1995 | Interferon maintenance therapy for patients with chronic lymphocytic leukemia in remission after fludarabine therapy | *Blood, 86*(4), 1298-1300. | Interferon |
| Zinzani, P. L.  1997 | Results of a fludarabine induction and alpha-interferon maintenance protocol in pretreated patients with chronic lymphocytic leukemia and low-grade non-Hodgkin's lymphoma. | *Eur J Haematol, 59*(2), 82-88. | Interferon |
| Mauro, F. R.  2003 | Fludarabine + prednisone +/- alpha-interferon followed or not by alpha-interferon maintenance therapy for previously untreated patients with chronic lymphocytic leukemia: long term results of a randomized study | *Haematologica, 88*(12), 1348-1357. | Interferon |
| Wendtner, C. M.  2004 | Consolidation with alemtuzumab in patients with chronic lymphocytic leukemia (CLL) in first remission--experience on safety and efficacy within a randomized multicenter phase III trial of the German CLL Study Group (GCLLSG). | *Leukemia, 18*(6), 1093-1101. | Alemtuzumab |
| Montillo, M.  2006 | Alemtuzumab as consolidation after a response to fludarabine is effective in purging residual disease in patients with chronic lymphocytic leukemia. | *J Clin Oncol, 24*(15), 2337-2342 | Alemtuzumab |
| Molica, S.  2007 | Intense reversal of bone marrow angiogenesis after sequential fludarabine-induction and alemtuzumab-consolidation therapy in advanced chronic lymphocytic leukemia. | *aematologica, 92*(10), 1367-1374 | Alemtuzumab |

Ongoing trials

| Autohrs/  Drugs | Title | Status |
| --- | --- | --- |
| NCT02758665 | A prospective, open-label, multicenter randomized phase-II trial to evaluate the efficacy and safety of a sequential regimen of obinutuzumab (Gazyvaro) followed by obinutuzumab and venetoclax, followed by either standard venetoclax maintenance or MRD guided venetoclax maintenance in first-line patients with CLL and unfit for FCRlike regimens | Active, not recruiting |
| NCT02401503 | Sequential Regimen of Bendamustine-Debulking Followed by ABT-199 and GA101-Induction and -Maintenance in CLL (CLL2-BAG) | Active, not recruiting |
| NCT03787264 | Sequential Regimen of Bendamustin-Debulking Followed by Obinutuzumab, Acalabrutinib and Venetoclax in Patients With Relapsed/Refractory CLL (CLL2-BAAG) | Recruiting |
| NCT03708003 | Ibrutinib lead-in Followed by Venetoclax Plus Ibrutinib in Patients With RR CLL | Recruiting |
| NCT02320383 | CLLR3: Bendamustine + GA101 (BG) in Relapsed or Refractory CLL Followed by GA101 Maintenance for Responding Patients | Active, not recruiting |
| NCT02388048 | Ofatumumab & Ibrutinib + Allogeneic Bone Marrow Transplant or Consolidation in High Risk Chronic Lymphocytic Leukemia | Recruiting |
| NCT00645606 | Rituximab Maintenance Versus Observation After First-line Immunochemotherapy by FCR in Older Patients With Chronic Lymphocytic Leukemia (LLC2007SA) | Completed,  Not publish |
| NCT01118234 | Rituximab Versus Observation as Maintenance Therapy in Chronic Lymphocytic Leukemia (Chronic Lymphocytic Leukemia) | Active, not recruiting |
| NCT01465334 | Ofatumumab With High Dose Methylprednisone Followed by Ofatumumab and Alemtuzumab in 17p CLL | Active, not recruiting |
| NCT03847727 | Bendamustine and Rituximab (BR) as Induction and Maintenance in Relapsed and Refractory Chronic Lymphocytic Leukemia | Active, not recruiting |
| NCT01754857 | Bendamustine and Rituximab Induction Therapy and Maintenance Rituximab and Lenalidomide in Previously Untreated CLL/SL | Recruiting |
| NCT01754870 | Phase II Study of Bendamustine and Rituximab Induction Chemoimmunotherapy Followed by Maintenance Rituximab (Rituxan®) and Lenalidomide (Revlimid®) in Relapsed and Refractory Chronic Lymphocytic Leukemia (CLL) and Small Lymphocytic Lymphoma (SLL) | Withdraw |
