# Supplementary figures and images for: A network meta-analysis of maintenance therapy in chronic lymphocytic leukemia
Source: PLoS One. 2020 Jan 29;15(1):e0226879. doi: 10.1371/journal.pone.0226879 (PMC6988939; doi:10.1371/journal.pone.0226879)

**S1 Figs**: Quality assessment (Cochrane risk of bias)


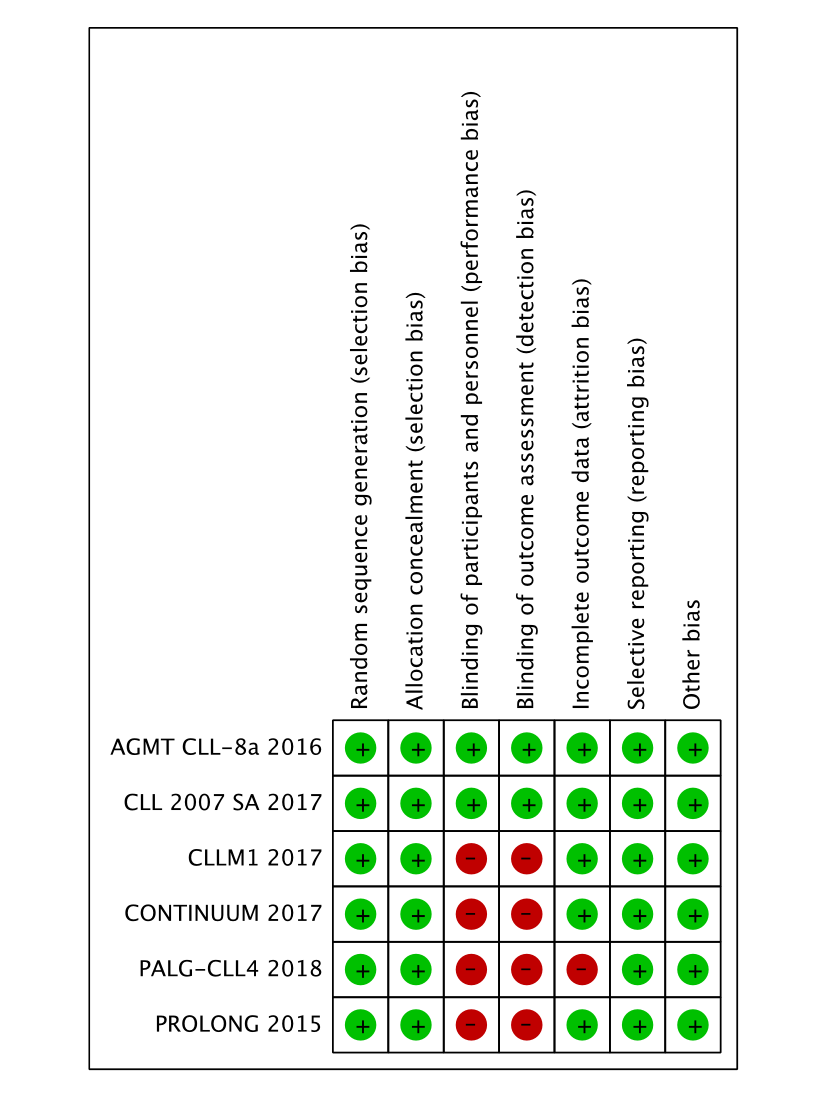

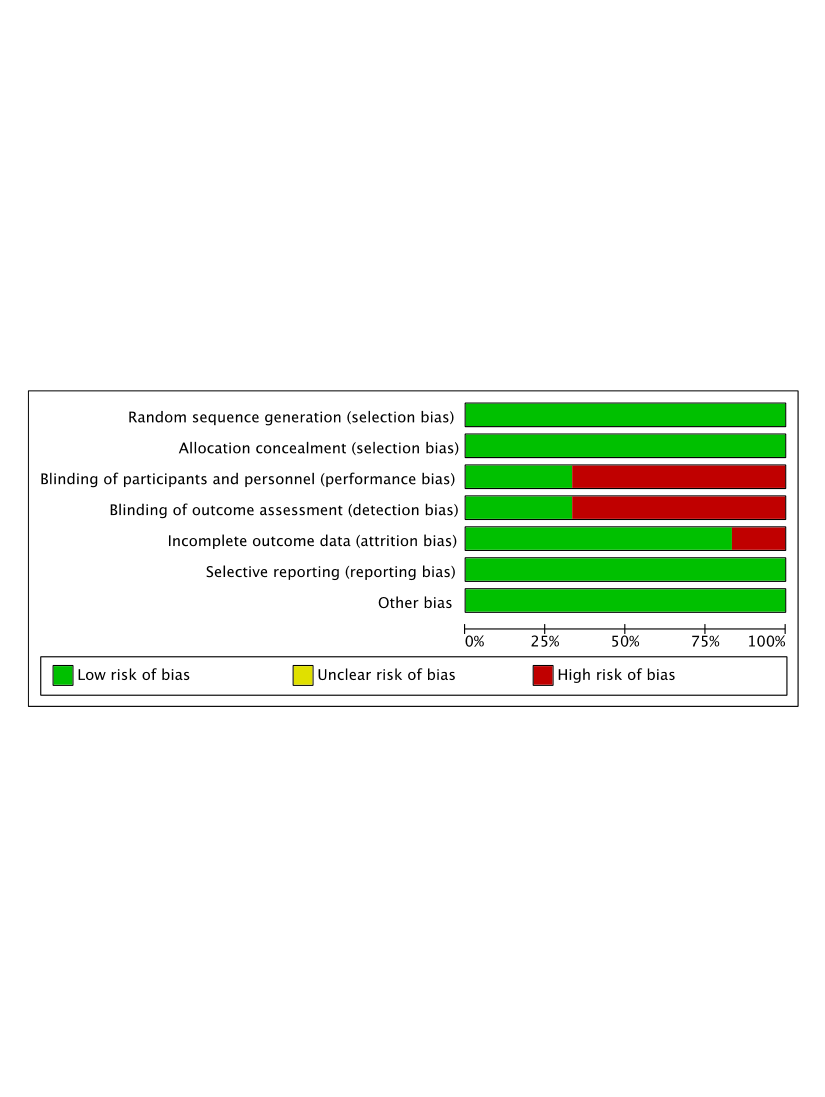

Supplement: S1 Fig — (DOCX) [file pone.0226879.s004.docx]

**S3 Figs** : Tabulated summary of average characteristics**
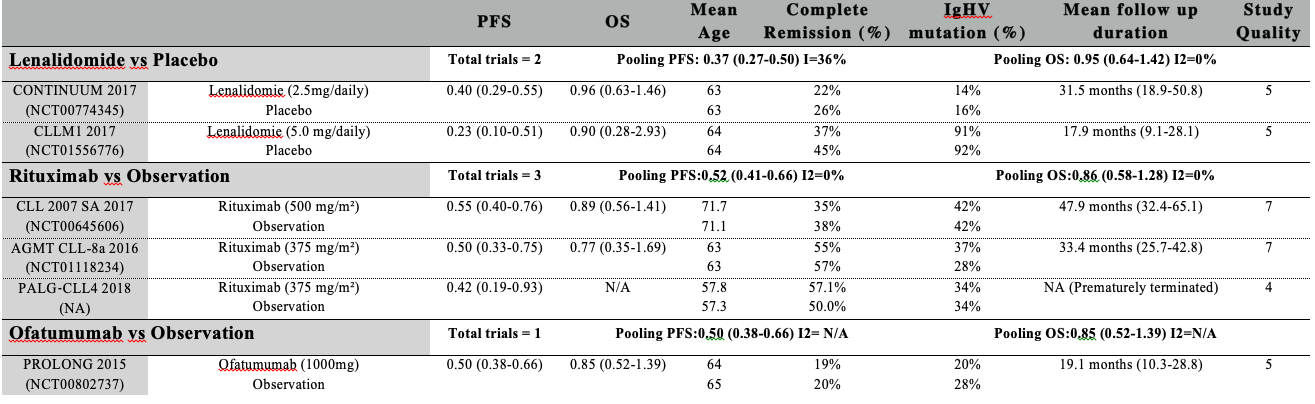
**

Supplement: S3 Fig — (DOCX) [file pone.0226879.s006.docx]
