# Supplementary material for: A network meta-analysis of maintenance therapy in chronic lymphocytic leukemia
Source: PLoS One. 2020 Jan 29;15(1):e0226879. doi: 10.1371/journal.pone.0226879 (PMC6988939; doi:10.1371/journal.pone.0226879)
Supplement: S2 Fig — (DOCX) [file pone.0226879.s005.docx]

**S2 figs:** Detail results of Pairwise Meta-analyses

| PFS (funnel plot) | OS(funnel plot) |
| --- | --- |
| 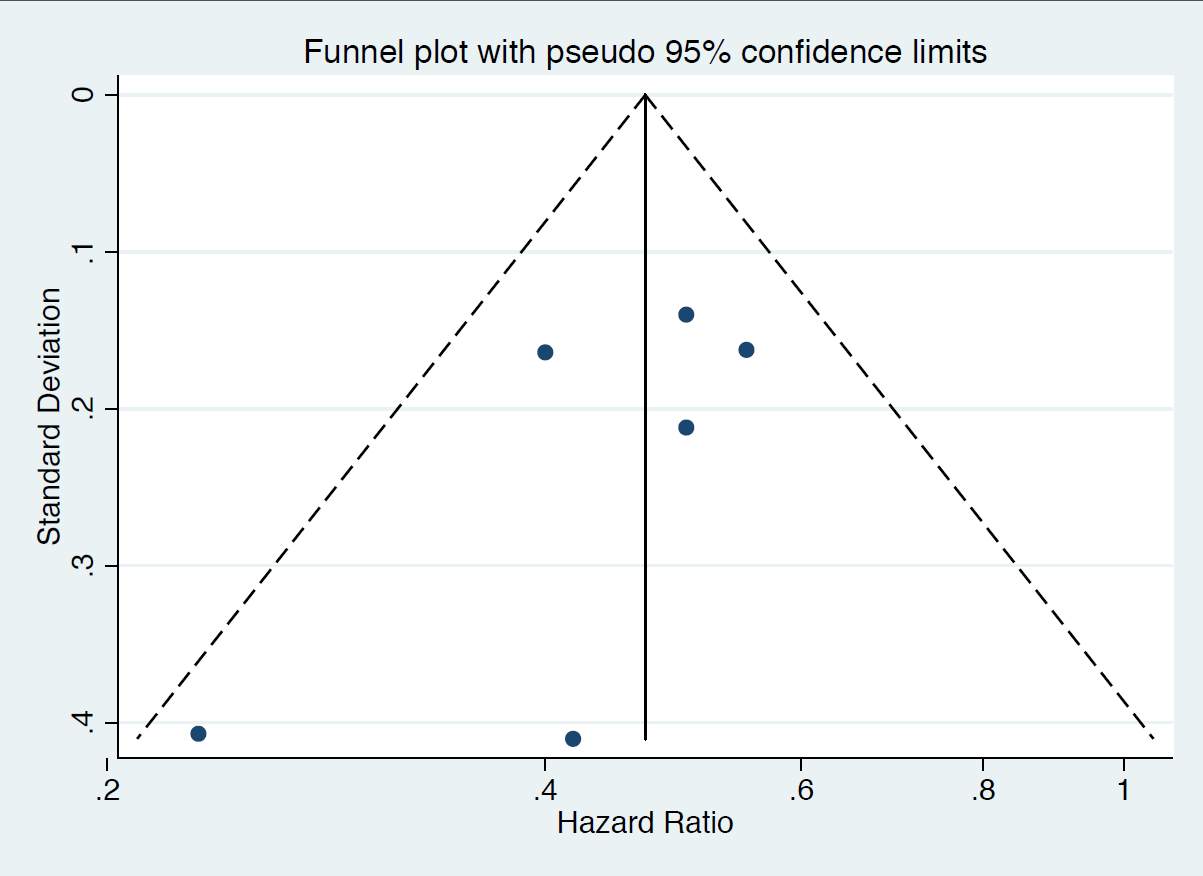 | 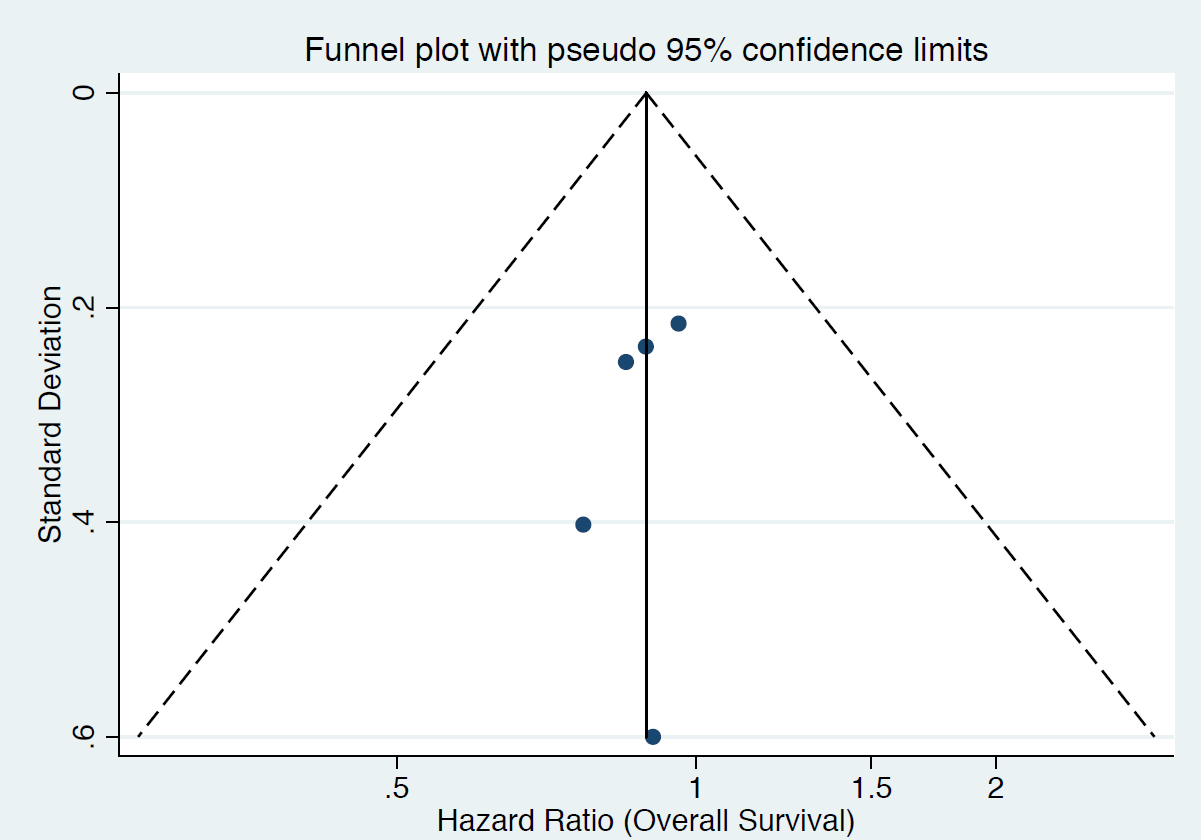 |
| SAE (forest plot) | SAE(funnel plot) |
|  | 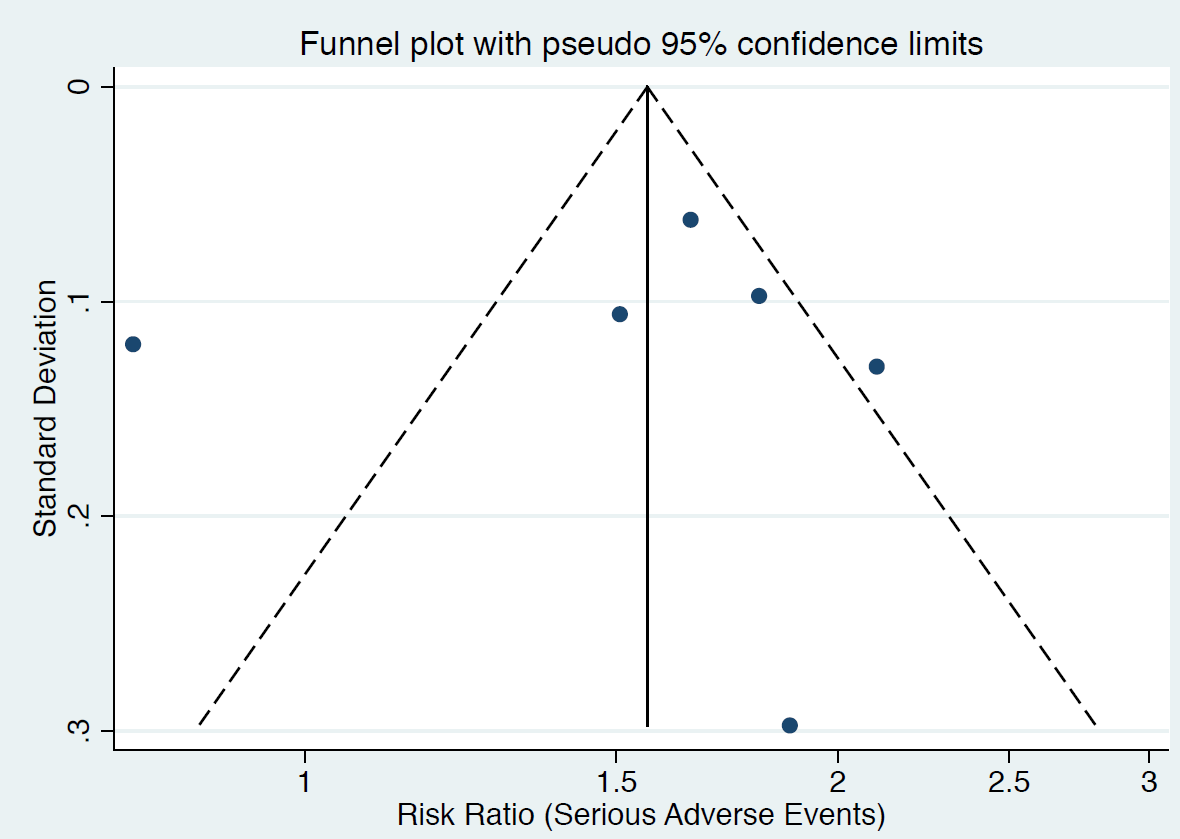 |
