# Supplementary material for: A network meta-analysis of maintenance therapy in chronic lymphocytic leukemia
Source: PLoS One. 2020 Jan 29;15(1):e0226879. doi: 10.1371/journal.pone.0226879 (PMC6988939; doi:10.1371/journal.pone.0226879)
Supplement: S4 Fig — (DOCX) [file pone.0226879.s007.docx]

**S4 figs**: Detail results of Network Meta-analyses

| 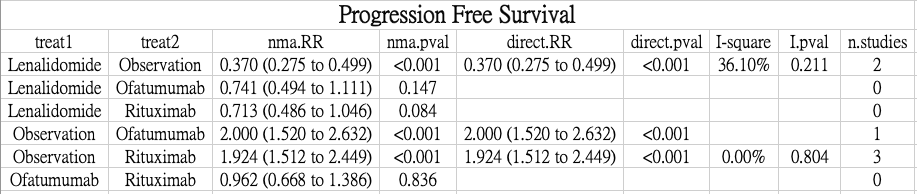 |
| --- |
| 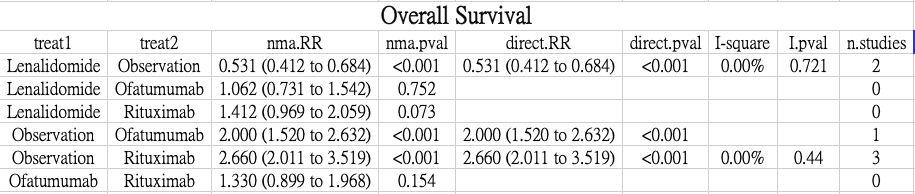 |
| 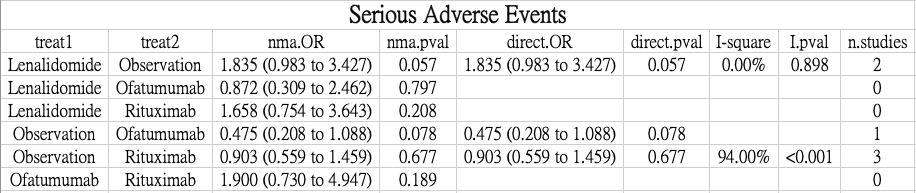 |
| 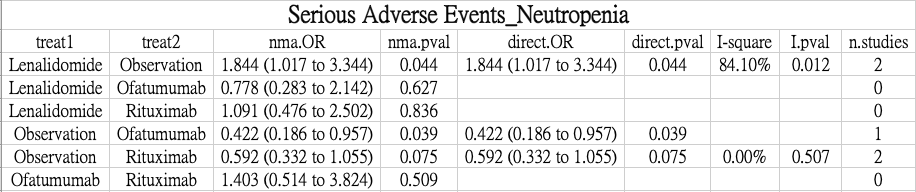 |
| 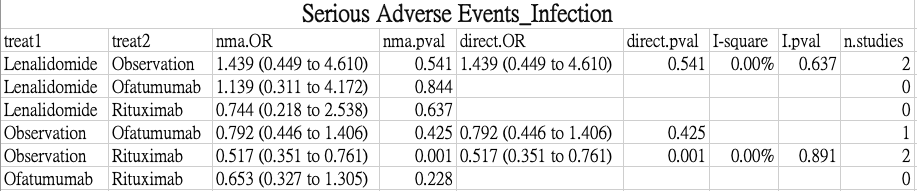 |

Comparison-adjusted funnel Plots

| PFS | OS |
| --- | --- |
| 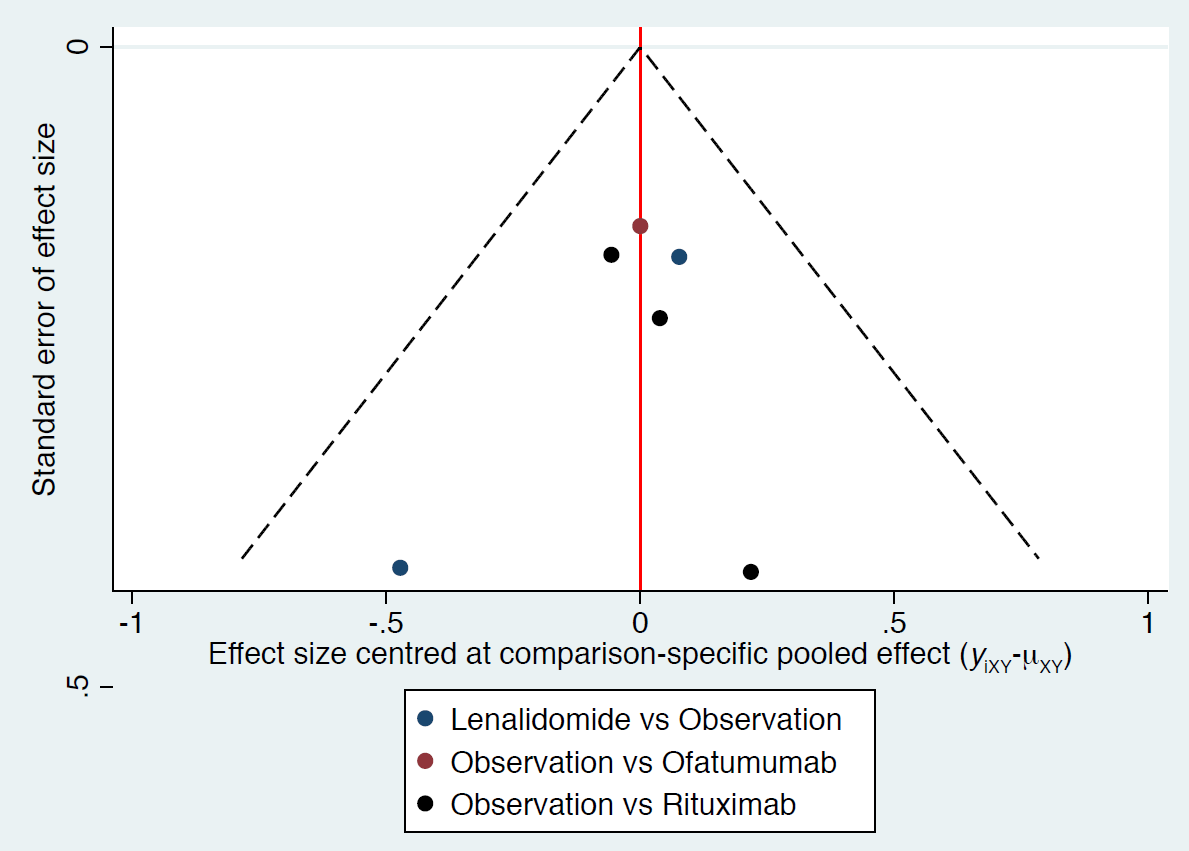 | 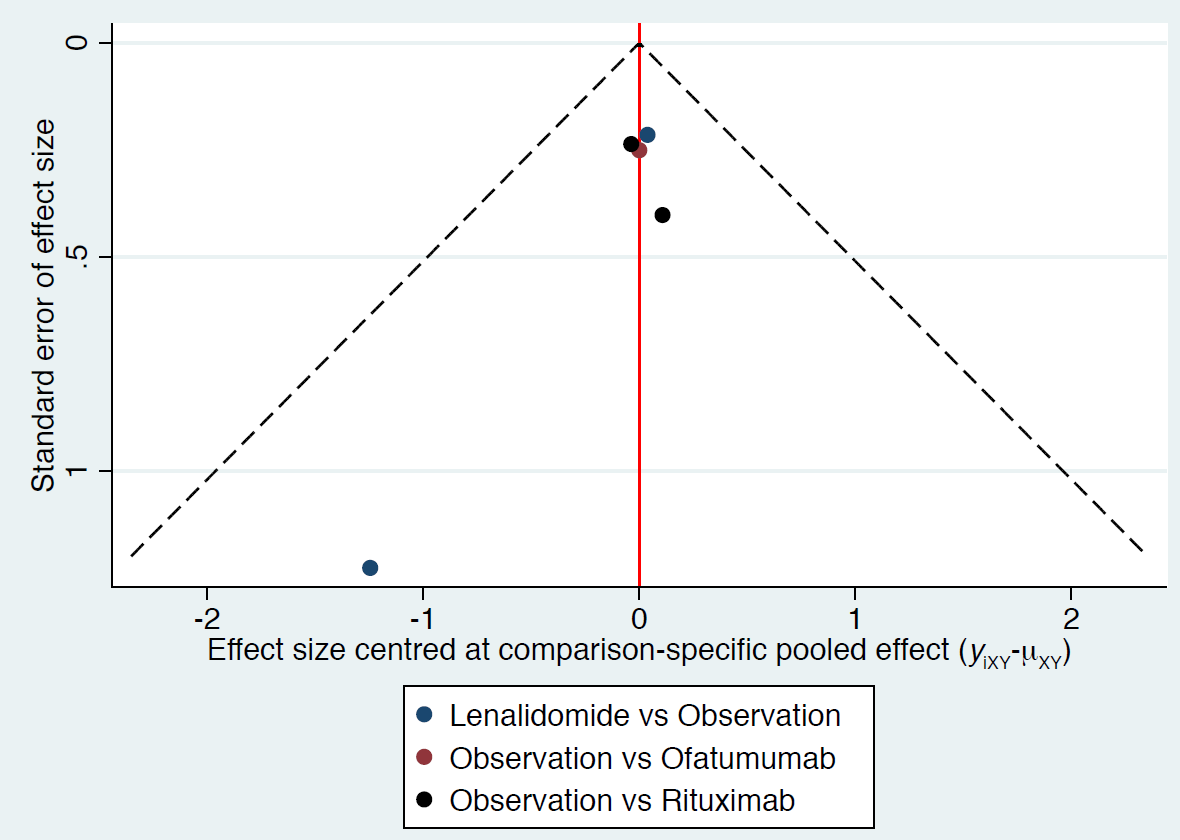 |

Contribution plot

| PFS | OS |
| --- | --- |
| 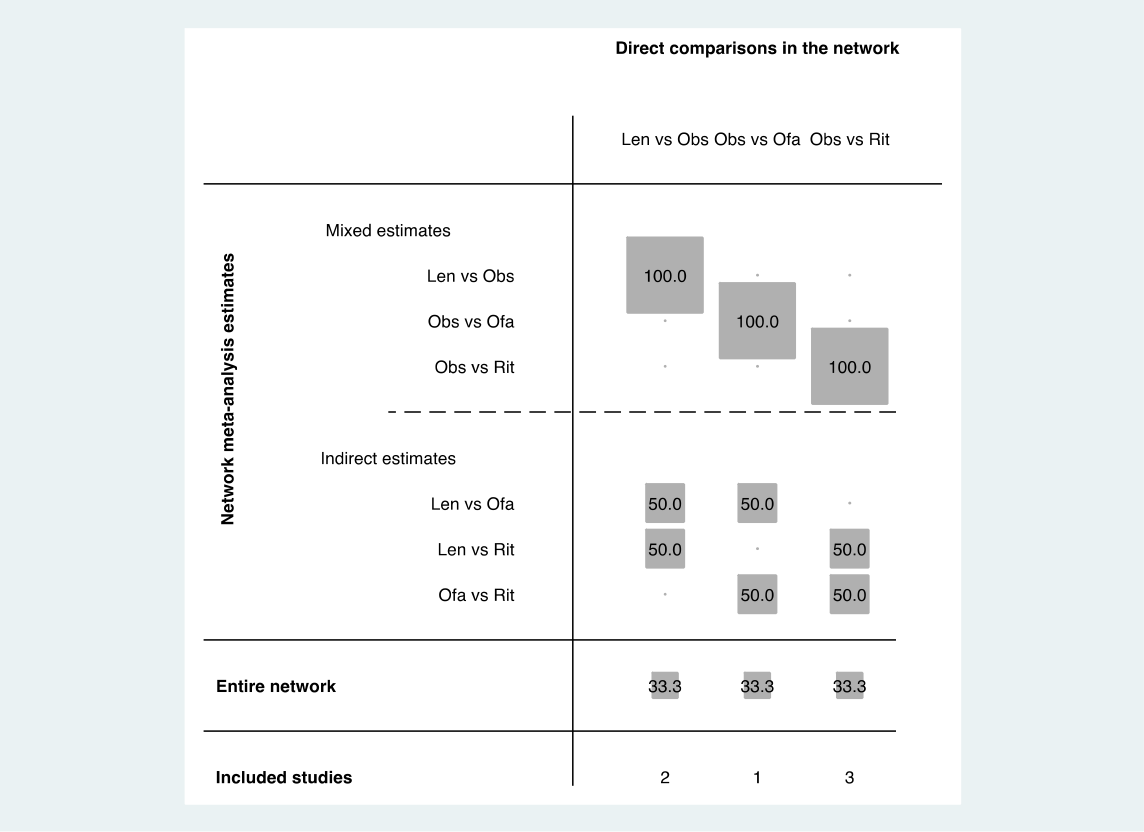 | 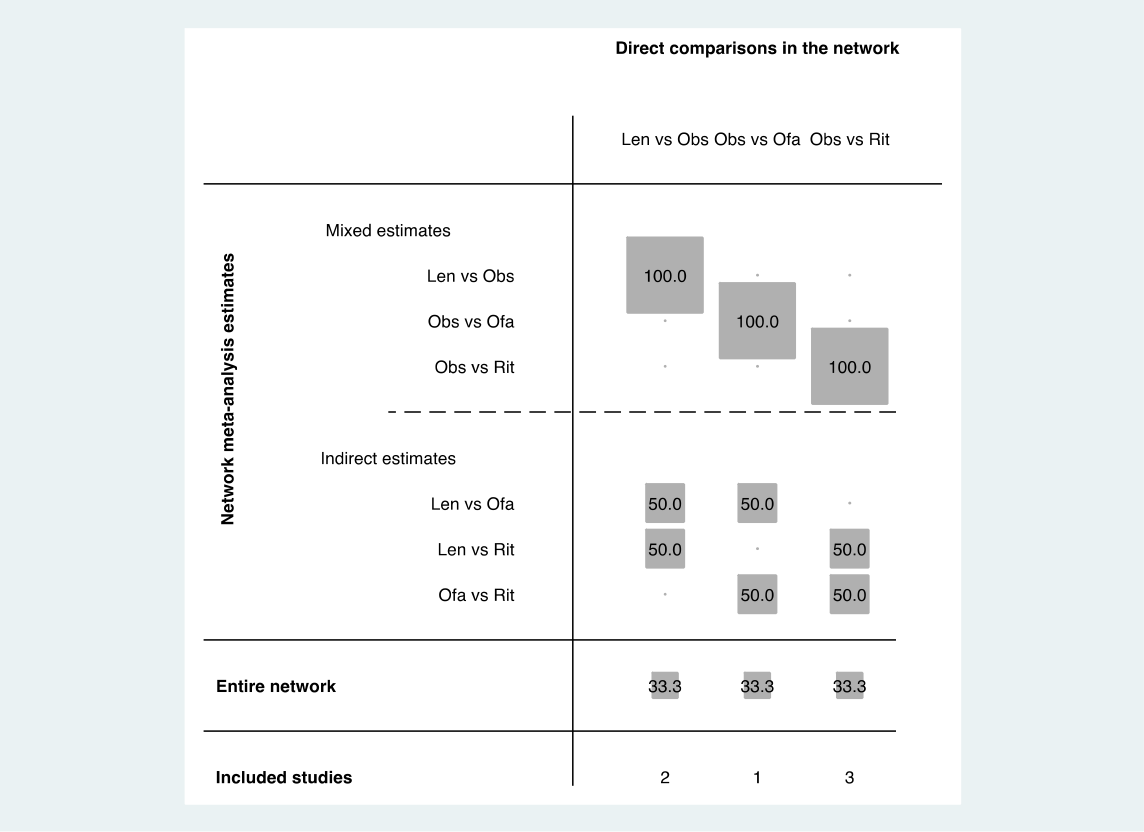 |
