# Supplementary material for: A network meta-analysis of maintenance therapy in chronic lymphocytic leukemia
Source: PLoS One. 2020 Jan 29;15(1):e0226879. doi: 10.1371/journal.pone.0226879 (PMC6988939; doi:10.1371/journal.pone.0226879)

**S5a Figs** : Subgroup Analysis of primary outcome (PFS)


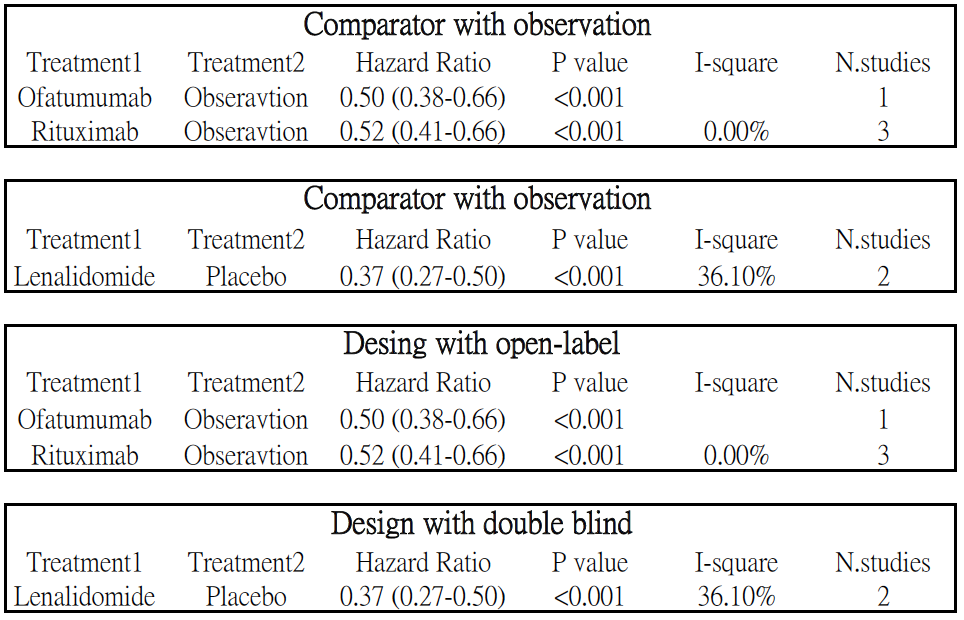


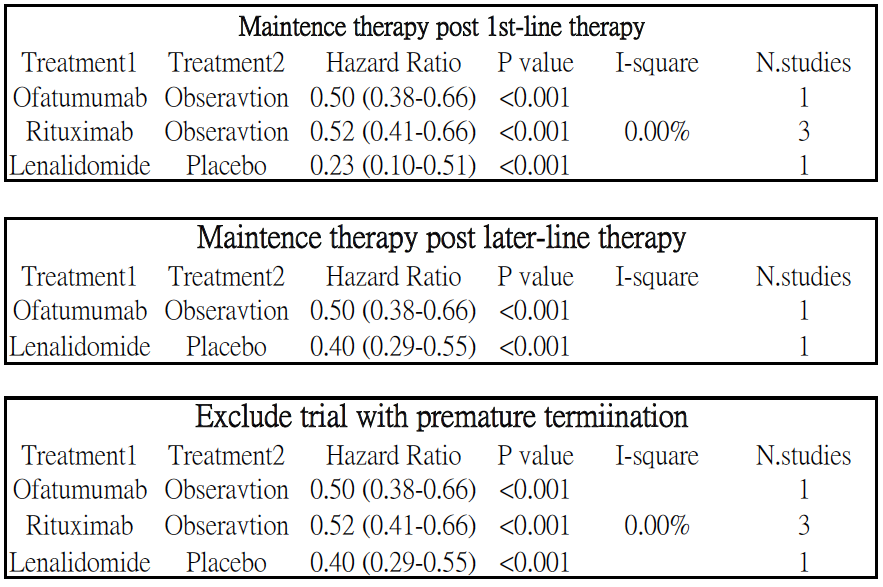


Exclude trial with premature termination

**S5a Figs** : Sensitivity Analysis of primary outcome (PFS)


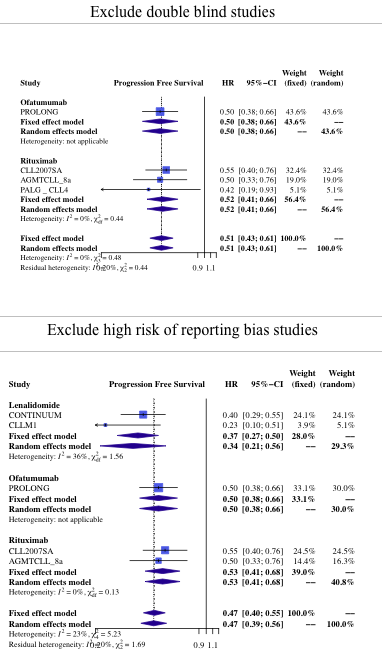

Supplement: S5 Fig — (DOCX) [file pone.0226879.s008.docx]
