# Supplementary material for: A network meta-analysis of maintenance therapy in chronic lymphocytic leukemia
Source: PLoS One. 2020 Jan 29;15(1):e0226879. doi: 10.1371/journal.pone.0226879 (PMC6988939; doi:10.1371/journal.pone.0226879)
Supplement: S6 Fig — (DOCX) [file pone.0226879.s009.docx]

**S6 Figs** : Network meta-analysis with random-effect model

| **** |
| --- |
| **** |
| **** |
